# A systematic review of factors associated with side‐effect expectations from medical interventions

**DOI:** 10.1111/hex.13059

**Published:** 2020-04-13

**Authors:** Louise E. Smith, Rebecca K. Webster, G. James Rubin

**Affiliations:** ^1^ Institute of Psychiatry, Psychology and Neuroscience King’s College London London UK; ^2^ Department of Psychology University of Sheffield Sheffield UK

**Keywords:** expectations, medications, nocebo effect, psychological predictors, side‐effects, symptoms

## Abstract

**Background:**

Fear of side‐effects can result in non‐adherence to medical interventions, such as medication and chemotherapy. Side‐effect expectations have been identified as strong predictors of later perception of side‐effects. However, research investigating predictors of side‐effect expectations is disparate.

**Objective:**

To identify factors associated with side‐effect expectations.

**Search strategy:**

We systematically searched Embase, Ovid MEDLINE, Global Health, PsycARTICLES, PsycINFO, Web of Science and Scopus.

**Inclusion criteria:**

Studies were included if they investigated associations between any predictive factor and expectations of side‐effects from any medical intervention.

**Data extraction and synthesis:**

We extracted information about participant characteristics, medication, rates of side‐effects expected and predictors of side‐effect expectations. Data were narratively synthesized.

**Main results:**

We identified sixty‐four citations, reporting on seventy‐two studies. Predictors fell into five categories: personal characteristics, clinical characteristics, psychological traits and state, presentation format of information, and information sources used. Using verbal risk descriptors (eg ‘common’) compared to numerical descriptors (eg percentages), having lower quality of life or well‐being, and currently experiencing symptoms were associated with increased side‐effect expectations.

**Discussion and conclusions:**

Decreasing unrealistic side‐effect expectations may lead to decreased experience of side‐effects and increased adherence to medical interventions. Widespread communications about medical interventions should describe the incidence of side‐effects numerically. Evidence suggests that clinicians should take particular care with patients with lower quality of life, who are currently experiencing symptoms and who have previously experienced symptoms from treatment. Further research should investigate different clinical populations and aim to quantify the impact of the media and social media on side‐effect expectations.

## 1 INTRODUCTION

Patients often fail to take medication as prescribed. Non‐adherence to prescribed treatments is thought to cost up to $52 000 (US$ 2015) per person annually worldwide.^1^ One of the main reasons why people do not take their medication is for fear of side‐effects.^2^, ^3^, ^4^ However, the cause of side‐effects attributed to medication is often unclear. While some may be directly caused by the medication, others may arise from the nocebo effect. This is a phenomenon whereby symptoms are attributed to an exposure, but they are not directly caused by the physical properties of the exposure. There is good evidence that expectation of symptoms from inert ‘placebo’ exposures such as sham pills, inhalers and odours can cause symptoms in those expecting them.^5^

Heightened side‐effect expectations are associated with later perception of side‐effects. Meta‐analytic results indicate that patient expectations for post‐chemotherapy side‐effects are associated with development of side‐effects from chemotherapy.^6^, ^7^, ^8^ Similarly, a prospective cohort study of parents vaccinating their child for influenza found that parents’ side‐effect expectations were the strongest predictor of parental report of side‐effects.^9^ Symptoms reported in the placebo arm of randomized placebo‐controlled trials may also arise from patient and investigator expectation.^10^, ^11^ Multiple systematic reviews and meta‐analyses have found similar rates and profiles of symptoms reported in the placebo and active drug arms of randomized placebo‐controlled trials across a range of medications.^10^, ^11^, ^12^, ^13^, ^14^, ^15^, ^16^, ^17^

There is little research investigating how side‐effect expectations develop. Beliefs about high dosage of the medication and explicit suggestions that the medication causes side‐effects may contribute to side‐effect expectations.^5^ How information about medical interventions, such as pharmacotherapy, chemotherapy and surgery, is framed by a health‐care professional or patient information leaflet may also affect side‐effect expectations.

Previous attempts to decrease side‐effect expectations and subsequent side‐effect experience include reducing information given to patients about potential side‐effects.^18^ This is problematic as it runs contrary to notions of informed consent and patient autonomy, and may breach laws ruling that information given to patients should not be ‘cherry picked’.^19^ Therefore, it is important to identify other factors that influence side‐effect expectations to provide alternative avenues for interventions which do not face this ethical issue.

The aim of this study was to provide an overview of the current literature on side‐effect expectations by conducting an exploratory systematic review to identify factors associated with expectations of more frequent side‐effects from medical interventions. We investigated psychological factors, identifying factors to target in interventions to reduce the nocebo effect, and personal and clinical factors, identifying populations who are particularly at risk of inaccurate expectations. Thus, results will provide us with two useful implications: how to minimize side‐effect expectations, and populations which may be particularly susceptible to heightened side‐effect expectations.

## 2 METHODS

We conducted a systematic review in accordance with PRISMA criteria^20^ to identify factors associated with expectations of side‐effects from medical interventions. We searched Embase, Ovid MEDLINE, Global Health, PsycARTICLES and PsycINFO through OvidSP, as well as searching Web of Science and Scopus. Our final search term was (symptom* OR side effect OR adverse effect OR adverse event OR adverse reaction) ADJ3 expect* (see Supporting Information S1). Databases were searched from inception to 6 March 2019. References and forward citations of included articles were also searched.

### 2.1 Inclusion criteria

Studies were included if they met the following criteria:

Participants: any age, or health status.

Predictors/exposures: investigated the association between psychological, social, contextual, or demographic factors and expectation that a medical intervention causes side‐effects (in an actual or hypothetical situation).

Outcome: expectation that any active medical intervention (eg pill, vaccine, asthma inhaler, chemotherapy, surgery) caused side‐effects. Studies investigating combined expectations about side‐effect frequency and severity were included; those which investigated only expectations about side‐effect severity were excluded. Studies investigating whether side‐effect expectations predicted later perception of side‐effects were excluded.

Study reporting: published in English. Studies were not excluded based on publication type.

### 2.2 Data extraction

We extracted information about study design, inclusion criteria, participant characteristics, medical intervention, rates of side‐effects expected and predictors of side‐effect expectations.

### 2.3 Risk of bias

Risk of bias was measured using an amended version of the Downs & Black checklist,^21^ a validated checklist,^22^ which is suitable for use in systematic reviews with appropriate modifications^23^ and which can be applied to reliably and validly evaluate randomized and non‐randomized studies, including observational studies using cross‐sectional and cohort methods.^24^ The modified version of this checklist has been used previously by our group.^4^, ^25^ The checklist evaluates studies on five dimensions: reporting (out of 10); external validity (out of two); internal validity—bias (out of three); confounding—selection bias (out of three); power (out of one). Scores were summed to give a total out of nineteen. Studies were rated as good quality if they scored a total of 16 or over; moderate quality if they scored 11‐15; and poor quality if they scored 10 or under. Studies were rated as poor quality for individual constructs if they scored: six or under for reporting; one or under for internal validity (bias), confounding (selection bias) and external validity; and if they did not include a justification for the sample size used.

LS and RW completed risk of bias ratings separately for 10 studies. Any discrepancies in scoring were discussed. LS and RW then completed ratings for 35 and 27 studies, respectively, which were cross‐checked by the other author. Any discrepancies were solved through discussion. Final scores were approved by both authors.

### 2.4 Procedure

LS came up with the search terms, carried out the search, screened papers, extracted data and completed risk of bias assessment. RW screened a random sample of 100 citations to full‐text screening stage, screened ten additional full texts and completed risk of bias assessment. Guidance was provided by GJR.

Data were narratively synthesized, taking study design and predictive validity into account when considering the strength of evidence for predictive factors. For psychological factors, experimental studies were considered to provide the strongest evidence, followed by longitudinal studies, then cross‐sectional studies. We counted cross‐sectional studies with factorial designs as experimental studies. For demographic characteristics which did not change, all study designs were considered equal.

## 3 RESULTS

### 3.1 Study characteristics

**FIGURE 1:**
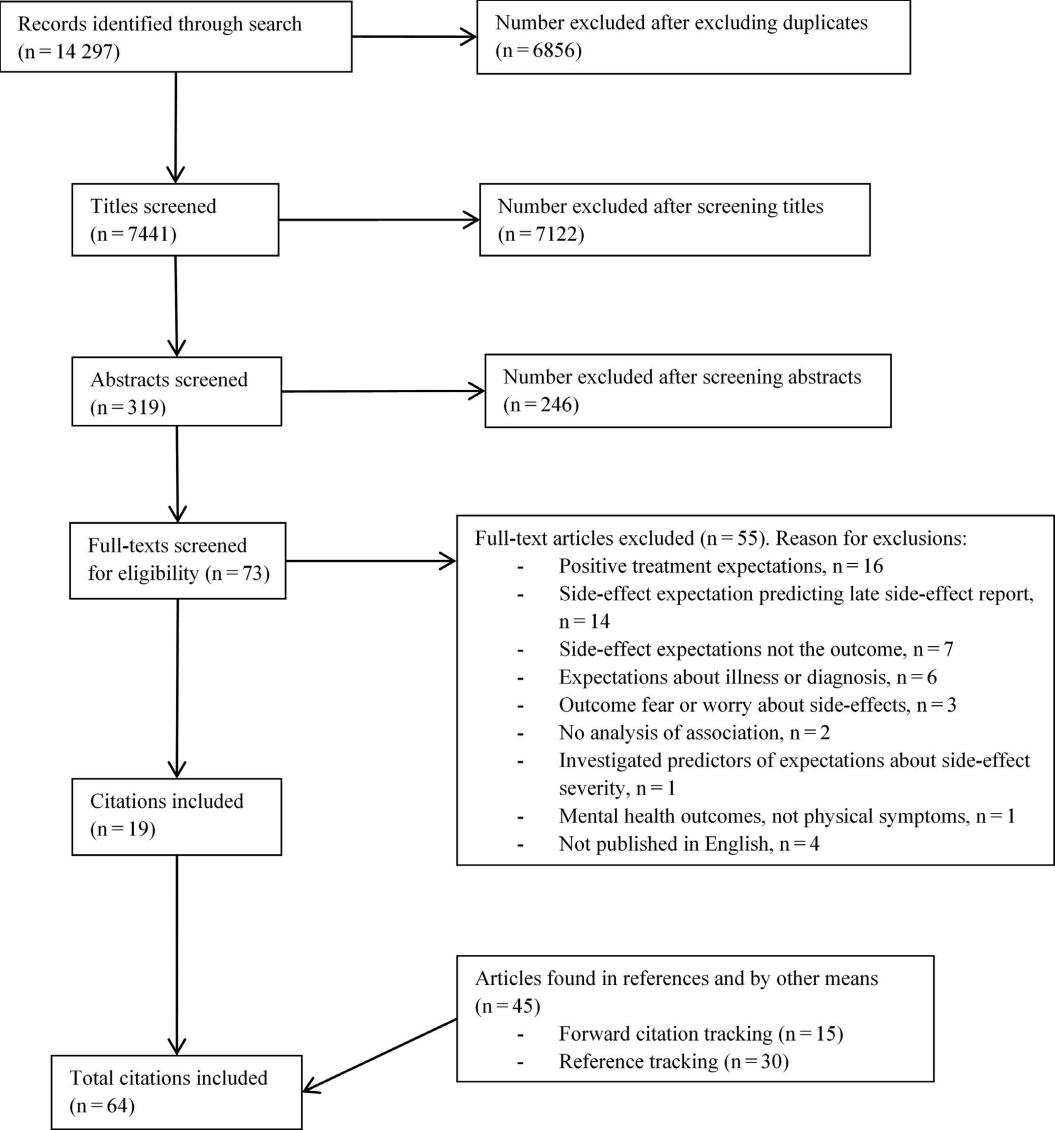
Flowchart depicting the selection of studies for the systematic review, with reasons for exclusion

A total of 14 297 citations were found by the original search. After removing duplicates, 7441 citations remained. After title, abstract and full‐text screening, nineteen citations remained. Forty‐five citations were identified by reference searching and forward citation tracking; none of these were found by the original search. Thus, 64 citations, reporting on 72 studies, met inclusion criteria (see Figure 1). Inter‐rater agreement for title, abstract and full‐text screening for the random sample of 100 citations was 100%; agreement for full‐text screening of ten additional full texts was also 100%.

Studies investigated side‐effect expectations for a range of medical interventions, including: chemotherapy; surgery; various medications including statins; and blood transfusion (for full list see Table 1).

**TABLE 1:** Methods of studies included in systematic review

Reference	Study design (method)	Number of participants (age)	Medication, actual or hypothetical situation	Inclusion criteria (location)	Risk of bias
Al Juffali et al 2014^26^	Cross‐sectional study, factorial design. Between groups, two factors: presentation format (verbal/numerical), side‐effect (dry eyes/loss of hair) (paper questionnaire)	141 (mean age 23 y, range 21 to 25 y)	Acne medication (roaccutane), actual situation	Patients who were newly prescribed Roaccutane for the first time at three selected hospitals (Saudi Arabia)	15
Andrykowski and Gregg 1992^27^	Prospective cohort study (paper questionnaire, medical records)	65 (mean age 59.6 y, SD = 11.7)	Chemotherapy, actual situation	Patients at a community‐based cancer centre who were at least 18 y old, and who received an initial course of intravenous cytotoxic chemotherapy as either an inpatient or outpatient, but were scheduled to receive their remaining chemotherapy infusions as an outpatient (not reported)	10
Berry et al 2002^28^; Berry et al 2002^29^	Study 1: Cross‐sectional study, factorial design. Between groups, two factors: side‐effect severity (mild/severe), response presentation format (percentage/frequency). Within groups, one factor: probability term (very common/common/uncommon/rare/very rare) (paper questionnaire)	268 (range 18 to 55 y)	Medication for throat or ear infections (counterbalanced across condition), hypothetical situation	Undergraduate and post‐graduate students who were members of the psychology department research panel (University of Reading, UK)	11
	Study 2: Cross‐sectional study, factorial design. Between groups, one factor: presentation format (verbal/numerical) (paper questionnaire)	112 (range 18 to 70 y)	Medication for throat or ear infections, hypothetical situation	Volunteers from the general population (Reading, UK)	10
Berry et al 2002^30^	Study 1: Cross‐sectional study, factorial design. Between groups, four factors: disease severity (mild/severe), side‐effect severity (mild/severe), side‐effect likelihood (likely/unlikely), side‐effect number (many/few) (paper questionnaire)	976 (range 18 to 70 y)	Medication for one of two diseases, hypothetical situation	Volunteers from the general population (Reading, UK)	10
	Study 2: Cross‐sectional study, factorial design. Between groups, three factors: disease severity (mild/severe), side‐effect severity (mild/severe), benefit statement (no statement/positive benefit/unknown benefit) (paper questionnaire)	592 (range 18 to 70 y)			9
	Study 3: Cross‐sectional study, factorial design. Between groups, three factors: disease severity (mild/severe), side‐effect severity (mild/severe), benefit statement (no statement/prevention statement/alleviation statement) (paper questionnaire)	515 (range 18 to 70 y)			9
Berry et al 2003^31^	Study 1 and 2: Cross‐sectional study, factorial design. Between groups, one factor: personalization (personalized/not‐personalized) (paper questionnaire)	Study 1: 95 (range 18 to 50 y)	Medication for pneumonia or another severe illness (not named), hypothetical situation	Volunteers from the general population (Reading, UK)	11
		Study 2: 100 (range 18 to 60 y)	Medication for subacute thyroiditis, hypothetical situation		11
Berry et al 2003^32^; Berry et al 2002^29^	Study 1: Cross‐sectional study, factorial design. Between groups, two factors: age (18‐40,41‐60,61‐80), presentation format (verbal/numerical) (paper questionnaire)	120 (18 to 40 y, n = 40; 41 to 60 y, n = 40; 61 to 80 y, n = 40)	Medication for throat or ear infection, hypothetical situation	Volunteers from the general population (Reading, UK)	10
	Study 2: Cross‐sectional study, factorial design. Between groups, three factors: presentation format (verbal/numerical), frequency (common/rare), side‐effect severity (severe/ mild) (paper questionnaire)	360 (18 to 75 y)			10
Berry 2004^33^	Cross‐sectional study, factorial design. Between groups, one factor: parental status (parent/not parent). Within groups, one factor: patient type (adult/child) (paper questionnaire)	136 (range 20 to 50 y)	Medication for throat or ear infection, hypothetical situation	Volunteers from the general population (Reading, UK)	10
Berry et al 2004^34^	Study 1: Cross‐sectional study, factorial design. Between groups, one factor: side‐effect severity (mild/severe). Within groups, one factor: probability term (very common/common/uncommon/rare/very rare). Compared with results from Berry et al 2002^28^ (paper questionnaire)	56 (mean age 37 y, range 28 to 55 y)	New short course antibiotic, hypothetical situation	Doctors attending a rheumatology conference (UK)	11
	Study 2: Cross‐sectional study, factorial design. Between groups, two factors: sample population (student/ doctor), side‐effect severity (mild/severe). Within groups, one factor: probability term (high >1%/moderate 0.1%–1%/low 0.01%–0.1%/very low 0.001%–0.0001%/minimal 0.0001%–0.00001%/ negligible <0.00001%) (paper questionnaire)	55 medical doctors (mean age 36 y, range 28 to 48 y), 160 students (mean age 29 y, range 19 to 50 y)	Doctors: new short course antibiotic, hypothetical situation Students: medication for throat or ear infection, hypothetical situation	Doctors: Doctors attending a rheumatology conference Students: undergraduate and post‐graduate students at the University of Reading (UK)	10
Berry et al 2004^35^	Cross‐sectional study, factorial design. Between groups, one factor: presentation format (verbal/numerical) (paper questionnaire)	188 (range 18 to 70 y)	Ibuprofen, hypothetical situation	Volunteers from the general population (Reading, UK)	13
Berry et al 2006^36^	Cross‐sectional study, factorial design. Between groups, two factors: risk increase format (relative risk, absolute risk, number needed to harm), baseline information (included, not included) (paper questionnaire)	268 (mean age 29 y, SD = 12.54, range 18 to 45 y)	Oral contraceptive pill, mixed—presented as hypothetical situation but 30% were currently taking pill and 37% had done so in the past	Female volunteers from the general population (Reading, UK)	12
Bersellini and Berry 2007^37^	Study 2: Cross‐sectional study, factorial design. Between groups, two factors: effectiveness statement (included/not included), rationale statement (included/not included) (paper questionnaire)	292 (range 18 to 75 y)	Short course antibiotic for a throat infection, hypothetical situation	Volunteers from the University of Reading (UK)	10
Blalock et al 2016^38^	Randomized trial, factorial design. Between groups, two factors: side‐effect probability format (low side‐effect probability, numeric/ high side‐effect probability, numeric/non‐numeric), benefit condition (low benefit probability, risk with and without treatment numeric/high benefit probability, risk with and without treatment numeric/low benefit probability, risk difference numeric/high benefit probability, risk difference numeric/non‐numeric) (online questionnaire)	999 (mean age 33.9 y (SD = 11.1)	Medication to lower cholesterol, hypothetical situation	People from Amazon Mechanical Turk (remote)	12
Colagiuri et al 2008^39^	Randomized control trial (interview, questionnaire)	671 (average age 53 y, range 25 to 90 y)	Chemotherapy, actual situation	Patients aged 18 y or over with any cancer diagnosis and were about to receive their first chemotherapy treatment (USA)	14
Cox 2019^40^	Study 2: Cross‐sectional study, factorial design. Between groups, two factors: adjective (rare/common), adverb (very/no adverb) (online questionnaire)	712 (25 to 29 y, 16.5%; 30 to 34 y, 17.4%; 35 to 39 y, 17.4%; 40 to 44 y, 16.2%; 45 to 49 y, 15.8%; and 50 to 55 y, 16.7%)	Pain reliever, hypothetical situation	People from Survey Sampling International (remote)	9
Davis 2007^41^	Cross‐sectional study (online questionnaire)	669 (age 18 to 34 y, 36.5%; age 35 to 64 y, 61.9%)	Three prescription drugs (allergy, cholesterol, insomnia), hypothetical situation	Sample of 3200 individuals from Syracuse University's Study Response Project (USA)	10
Fischer and Jungermann 1996^42^	Study 1: Cross‐sectional study, factorial design. Between groups, one factor: presentation format (verbal/verbal and base rate). Within‐group: severity of side‐effect (mild/severe) (paper questionnaire)	82 (not reported)	Drug (disease not specified), hypothetical situation	Students from the Department of Psychology of the Technical University Berlin	8
Franic and Pathak 2000^43^	Cross‐sectionals study, mixed factorial design. Between groups, one factor: phrasing (second‐/third‐person description of scenarios). Within‐group: three factors: frequency of side‐effects (rarely/occasionally/frequently), severity of side‐effect (mild/severe), context (general/specific/very specific) (postal questionnaire)	74 (second person phrased surveys: mean age 28 y, range 19 to 41 y; third person phrased surveys: mean age 30, range 21 to 40 y)	General context: ‘new medication’, hypothetical situation Specific context: ‘new birth control pill, taken once daily at the same time each day’, hypothetical situation Very specific context: ‘new pain relief medication for period cramps (menstrual pain) which started at the earliest onset of pain and is taken every six hours when needed for pain relief. Patients typically require the pain killer for approximately three to five days’, hypothetical situation	Random sample of 400 (/842) female patients of child‐bearing age from the Women's Clinic at the Ohio State University Medical Center in Columbus (Ohio, USA)	13
Gardner et al 2011^44^	Cross‐sectional study, amalgamation of data from four separate studies including Knapp et al 2009^45^ and Knapp et al 2010^46^ (two studies not published), factorial designs (online questionnaires)	591 (mean age 46.5 y, SD = 10.8, range 15 to 66 y)	Tamoxifen (endocrine therapy for cancer), mixed—presented as hypothetical situation but 78% had cancer (n = 461), 44.3% were taking tamoxifen or had previously taken tamoxifen (n = 262)	People navigating to the Tamoxifen page on the www.cancerhelp.org.uk website (remote)	11
Goetsch et al 1991^47^	Prospective cohort study: baseline, one month follow‐up, three month follow‐up (postal questionnaires)	38, n = 19 taking contraceptives for the first time, n = 17 using oral contraceptives for at least 6 months (range 18 to 24 y)	Oral contraceptives, actual situation	Unmarried females recruited from contraceptive education programmes (West Virginia University and Western Michigan University, USA)	7
Heisig et al 2015^48^	Study 1 and 2: Cross‐sectional study using two time points (pre‐/post‐information), factorial design. Between groups, two factors: treatment benefit framing (no emphasis/emphasis), presentation (personalized/standard business‐like interaction) (not reported)	Study 1: 60 (mean age 50 y)	Adjuvant endocrine treatment for breast cancer, hypothetical situation	Healthy women older than 18 y who were fluent in German, without a history of mamma carcinoma, any other cancer diagnoses within the last 5 y, personal experience with endocrine treatment, or presence of a serious physical illness (not reported)	13
		Study 2: 64 (mean age 50 y)	Chemotherapy for breast cancer, hypothetical situation	Healthy women older than 18 y who were fluent in German, without a history of mamma carcinoma, any other cancer diagnoses within the last 5 y, personal experience with chemotherapy, or presence of a serious physical illness (not reported)	13
Heisig et al 2016^49^	Cross‐sectional study (not reported)	165 (mean age 58 y, SD = 9.59)	Adjuvant endocrine therapy for treatment of breast cancer, actual situation	Women with a diagnosis of hormone receptor‐positive breast cancer with an indication for adjuvant endocrine therapy, with no progress of disease or relapse, diagnosis of other current carcinoma, having received neo‐adjuvant chemotherapy, having a severe acute psychiatric disorder or a physical comorbidity substantially influencing quality of life, and starting endocrine therapy before baseline assessment or having pre‐experiences with endocrine therapy (four breast cancer centres in Marburg and Hamburg, Germany)	14
Hickok et al 2001^50^	Prospective cohort study (paper questionnaire)	63 (mean age 52.5 y, range 33 to 83 y)	Chemotherapy without concurrent radiotherapy, actual situation	Patients at the University of Rochester Cancer Center, two local affiliated hospitals and a private oncology practice in Rochester, NY, between December 1994 and September 1997 who were being treated with an initial course of chemotherapy, were not receiving radiotherapy concurrently, did not have any primary malignancy or metastatic disease affecting the brain, and were at least 19 y of age (New York, USA)	10
Hofman et al 2004^51^	Prospective longitudinal study (semi‐structured clinical interview, self‐report questionnaires, medical records)	938 (mean age 58 y, range 24 to 88 y)	Chemotherapy (n = 616, 66%) or radiation therapy (n = 538, 57%) for treatment of breast, lung, prostate, haematologic, gastrointestinal, or head and neck cancer (~25% patients received chemotherapy and radiation therapy), actual situation	Clinical outpatients at private medical oncology practices who were grantees of the National Cancer Institute's Community Clinical Oncology Program (CCOP) and were members of the University of Rochester Cancer Center (URCC) CCOP Research Base. Participants were newly diagnosed with cancer whose treatment plan included chemotherapy or radiation who had not had previous chemotherapy or radiation therapy (USA)	13
Hofman et al 2004^52^	Cross‐sectional study (questionnaire)	1015 (not reported)	Chemotherapy and radiation treatment, actual situation	Cancer patients from 17 Community Clinical Oncology Program (CCOP) institutions affiliated with the University of Rochester CCOP research base USA)	4
Hubal and Day 2006^53^	Cross‐sectional study. Frequency experiment. Within groups, one factor: side‐effect frequency terms (38 terms used). Severity experiment. Within groups, one factor: severity term (19 terms used) (questionnaire)	222. Numeric task, n = 206; visual task, n = 16 (not reported)	Side‐effects (medication not reported), hypothetical situation	Undergraduate students from Duke University (USA)	7
Jacobsen et al 1993^54^, ^a^	Prospective cohort study (in person and telephone interviews)	53 (mean age 49 y, range 29 to 78 y)	Adjuvant chemotherapy treatment for breast cancer, actual situation	Patients at a cancer centre who were aged 18 y or over, had undergone mastectomy for breast carcinoma, had not previously received radiotherapy or cytotoxic chemotherapy and who were scheduled to receive their first six infusions of outpatient adjuvant chemotherapy (not reported)	8
Montgomery et al 1998^55^, ^a^	Prospective cohort study (in person and telephone interviews)	59 (mean age 48.8 y)	Adjuvant chemotherapy treatment for breast cancer (sixth infusion), actual situation	Women diagnosed with Stage I or II breast cancer who were at least 18 y of age, had undergone mastectomy, had completed standard nurse‐administered pre‐chemotherapy teaching and had not previously received radiotherapy or cytotoxic chemotherapy (not reported)	
Knapp et al 2001^56^	Cross‐sectional study, factorial design. Between groups, two factors: presentation format (verbal/numerical), frequency of side‐effect (very common/common/uncommon/rare) (paper questionnaire)	155 (38% aged over 40 y)	Asthma, antibiotics, statins, actual situation	Adult attenders at a community pharmacy; a general practice asthma clinic and hospital cardiac rehabilitation clinic, who were on inhalers for asthma, antibiotics or a statin (not reported)	7
Knapp et al 2004^57^	Randomized controlled trial. Between groups, two factors: presentation format (verbal/numerical), side‐effect (constipation/pancreatitis) (paper questionnaire)	120 (median age 63 y, range 35‐74 y)	Simvastatin or atorvastatin, actual situation	Adults attending cardiac rehabilitation clinics at two hospitals following a recent admission for coronary artery bypass surgery or myocardial infarction who were taking either simvastatin or atorvastatin (Leeds, UK)	14
Knapp et al 2009^58^	Cross‐sectional study, factorial design. Between groups, one factor: presentation format (verbal/percentage/frequency) (online questionnaire)	148 (mean age 42.9 y, SD = 12.8, range 18 to 66 y)	Study 1: Taxol ® (chemotherapy treatment), mixed—presented as hypothetical situation but some participants had had side‐effects from Taxol ® before	People navigating to Taxol^®^ page on the www.cancerhelp.org.uk website (remote)	10
	Cross‐sectional study, factorial design. Between groups, one factor: presentation format (verbal/percentage/frequency) (online questionnaire). Within‐group, one factor: response format (percentage/frequency) (online questionnaire)	137 (mean age 39.1 y, SD = 14.2, range 16 to 66 y)	Study 2: Ibuprofen, mixed—presented as hypothetical situation but some participants had had side‐effects from ibuprofen before	People navigating to a page on pain management on the www.cancerhelp.org.uk website (remote)	10
Knapp et al 2009^45^	Cross‐sectional study, factorial design. Between groups, one factor: presentation format (verbal/frequency/combined) (online questionnaire)	187 (mean age 42.8 y, SD = 12.9, range 15 to 66 y)	Tamoxifen (endocrine treatment for cancer), likely to be mixed, but presented as hypothetical situation (not reported if some participants were taking/had taken tamoxifen	People navigating to the Tamoxifen page on the www.cancerhelp.org.uk website (remote)	14
Knapp et al 2010^46^	Cross‐sectional study, factorial design. Between groups, two factors: type of numerical description (absolute frequency/frequency band), presentation format (numerical/combined) (online questionnaire)	134 (mean age 47.6 y, SD = 9.1)	Tamoxifen (endocrine therapy for cancer), mixed—presented as hypothetical situation but 48.6% currently taking tamoxifen, 4.5% previously taken tamoxifen, 20.2% about to take tamoxifen	People navigating to the www.cancerhelp.org.uk website (remote)	10
Knapp et al 2013^59^	Randomized controlled trial. Three presentation formats: frequency, percentage, combined (online questionnaire)	129 (mean age 49.2 y, SD = 9.6, range 15 to 66 y)	Tamoxifen (endocrine therapy for cancer), mixed—presented as hypothetical situation but 51.9% currently taking tamoxifen, 11.6% previously taken tamoxifen, 20.2% about to take tamoxifen	People navigating to the Tamoxifen page on the www.cancerhelp.org.uk website (remote)	16
Knapp et al 2016^60^	Cross‐sectional study, factorial design. Between group, two factors: presentation format (numerical/verbal and numerical), verbal qualifier (may affect/will affect) (online questionnaire)	339 (mean 48.5 y, range 16 to 80 y)	Paclitaxel (Taxol; chemotherapy treatment), mixed—presented as hypothetical situation but 7.7% currently taking Taxol, 6.1% previously taken Taxol, 3.9% about to take Taxol	People navigating to the webpage on drugs commonly used in the treatment of cancer or to the webpage on Taxol on the www.cancerhelp.org.uk website (remote)	16
Lynch and Berry 2007^61^	Study 1: Cross‐sectional study, factorial design. Within groups, one factor: medicine type (prescribed/over‐the‐counter/herbal) (paper questionnaire)	77 (range 18 to 70 y)	Medication for persistent stomach indigestion, hypothetical situation	Volunteers from the general population (UK)	13
Mapes 1979^62^	Cross‐sectional study. Between groups, one factor: drug (beta‐blocker/antihistamine and chloramphenicol/neomycin sulphate) (postal questionnaire)	64 (not reported)	Beta‐blocker, antihistamine, chloramphenicol, neomycin sulphate, hypothetical situation	Two groups of male unrestricted principals in general practice who were physicians (East Anglia, UK)	4
Mazur and Merz 1994^63^	Cross‐sectional study, factorial design. Between groups, two factors: scale length (long/short), severity of complication (death from anaesthesia/severe pneumonia) (online questionnaire)	210 (not reported)	Operation, hypothetical situation	Patients in a general medical clinic seen consecutively for their medical problems by the physician investigator in a university‐based Department of Veterans Affairs Medical Center (not reported)	8
Montgomery and Bovbjerg 2003^64^	Prospective cohort study (paper questionnaire)	80 (mean age 6.9 y)	Adjuvant chemotherapy treatment for breast cancer, actual situation	Patients at an outpatient breast cancer centre who were at least 18 y of age; had never previously received radiation therapy or cytotoxic chemotherapy; had been diagnosed with Stage I or II breast cancer; were status post‐radical, modified radical, or segmental mastectomy; and were scheduled to receive outpatient adjuvant chemotherapy every 21 d (not reported)	11
Montgomery and Bovbjerg 2004^65^	Prospective cohort study (paper questionnaire)	63 (mean age 48.7, SD = 12.4)	Surgery for breast cancer (lumpectomy, excisional breast biopsy), actual situation	Patients scheduled for breast cancer surgery (not reported)	9
Moraes and Dal Pizzol 2018^66^	Randomized controlled trial, three presentation format: verbal descriptor and percentage range, percentage range, absolute percentage (paper questionnaire)	389 (18 to 34 y, n = 32; 35 to 59 y, n = 152; ≥60 y, n = 205)	Medicine for gastrointestinal problems, hypothetical situation	People aged over 18 with normal cognitive and communication skills who went to a ‘training pharmacy’ (enables internship training for pharmacy students) located in a university (Brazil)	18
O’Connor et al 1996^67^; O’Connor et al 1997^68^	Prospective cohort study (self‐report questionnaire and telephone interview)	292 (positive framing condition: mean age 53 y, SD = 13; negative framing condition, mean age 52 y, SD = 14)	Influenza vaccine, mixed—presented as hypothetical situation but uptake of the vaccine was measured as one of the study outcomes	Patients from outpatient respiratory and cardiac clinics at two teaching hospitals and one private group respiratory practice who were recommended for influenza immunization, that is were aged 65 y or over, or under 65 with chronic pulmonary or cardiac disorders severe enough to require regular medical follow‐up or hospital care (Ottawa, Canada)	17
Ohnishi et al 2002^69^	Cross‐sectional study (paper questionnaire)	168 patients (mean age 51 y, SD = 18.1, range 17 to 83 y) 156 physicians (mean age 36 y, SD = 8.2 range 24 to 76 y)	Cold medicine, anti‐cancer drug, hypothetical situation	Japanese patients aged 16 or over at the General Medicine Clinic at Kyoto University Hospital with no moderate or severe distress or cognitive problems and physicians from the Japanese General Medicine Research Network (Japan)	11
Pan et al 2018^70^	Prospective cohort study (semi‐structured interview, clinical assessment, medical records, questionnaire)	116 (mean age 55.4 y, SD = 9.97)	Adjuvant endocrine treatment for breast cancer, actual situation	Women with hormone receptor‐positive breast cancer or ductal carcinoma in situ indicated for adjuvant endocrine therapy	11
Parrella et al 2013^71^	Cross‐sectional study (computer aided telephone interviews)	469 (age 18 to 34 y, n = 89; age 35 to 44 y, n = 220; age 45 y and over, n = 160)	Immunizations, hypothetical situation	Adults who were randomly selected from electronic residential telephone listings who identified as a parent or legal guardian of children aged 18 y or younger (rural and metropolitan South Australia)	17
Roscoe et al 2000^72^	Prospective cohort study (questionnaire)	Study 1: 29 (mean age 60.5 y, SD = 11.4, range 34 to 79 y)	Chemotherapy for ovarian cancer, actual situation	Women with ovarian cancer who were being treated with either cisplatin or carboplatin as inpatients at the University of Rochester Cancer Center who were chemotherapy naïve (New York, USA)	12
		Study 2: 81 (mean age 54.1 y, SD = 11.8, range 33 to 83)	Chemotherapy for a variety of cancer diagnoses, actual situation	Patients with a variety of cancer diagnoses being treated with a variety of chemotherapy drugs at the University of Rochester Cancer Center, two locally affiliated hospitals, and a private oncology practice in Rochester who were chemotherapy naïve (New York, USA)	12
Schnur et al 2007^73^	Cross‐sectional study (paper questionnaire)	418 (mean age 48.3 y, SD = 13.7, range 19 to 83 y)	Breast conserving surgery, actual situation	Female patients scheduled for breast conserving surgery by two surgeons who were at least 18 y old and who were not currently in treatment for a psychiatric illness (New York, USA)	12
Schwartz et al 2009^74^	Randomized controlled trial—symptom drug box (telephone interview, postal questionnaire)	Control group, n = 109 (mean age 53 y, interquartile range 43 to 63 y). Drug box group, n = 122 (mean age 55 y, interquartile range 47 to 61 y).	Drug for heartburn (proton‐pump inhibitor and histamine‐2 blocker), hypothetical situation	Volunteers who were aged 35 to 70 y from a random‐digit dial sample of 3000 (USA)	8
	Randomized controlled trial—prevention drug box (telephone interview, postal questionnaire)	Control group, n = 108 (mean age 55 y, interquartile range 47 to 62 y). Drug box group, n = 111 (mean age 54 y, interquartile range 47 to 60 y).	Statins and clopidogrel for secondary cardiovascular prevention, hypothetical situation	Volunteers who were aged 35 to 70 y from a random‐digit dial sample of 3000 (USA)	8
Shedden‐Mora et al 2017^75^	Prospective cohort study, randomized controlled trial (not reported)	196 (not reported)	Adjuvant endocrine therapy for breast cancer, actual situation	Women with breast cancer (not reported)	2
Shelke et al 2008^76^	Randomized trial, two groups: standard education materials (control), standard education materials plus supplement about effectiveness of medication at controlling nausea and vomiting (intervention) (paper questionnaire)	358 (control group, mean age 57.8 y, SD = 13.4, range 28.3 to 91.4 y. Intervention group, mean age 57.4 y, SD = 12.1, range 27.4 to 84.3 y)	Chemotherapy, actual situation	Chemotherapy‐naïve cancer patients scheduled to receive their first treatment at eighteen private medical oncology practice groups that were grantees of the National Cancer Institute's Community Clinical Oncology Program (CCOP) and were members of the University of Rochester Cancer Center CCOP Research Base (USA)	13
Sullivan et al 2015^77^	Benefit design study. Cross‐sectional study, factorial design. Between groups, two factors: benefit (low/high), presentation format (numeric/numeric and qualitative/absolute difference and qualitative/full) and no‐information and qualitative only (online questionnaire)	2537 (18 to 40 y, n = 594; 41 to 52 y, n = 639; 53 to 64 y, n = 683; 65 + y, n = 621)	Bone loss from fictitious prescription drug for heartburn, hypothetical situation	Online panellists from the 2007 National Health Interview Survey data with self‐reported symptoms of heartburn or acid reflux in the last 3 mo, who were aged 18 or over, and were not working for a pharmaceutical, advertising, or market research company; and not being a health‐care professional (remote)	15
	Risk design study. Cross‐sectional study, factorial design. Between groups, two factors: risk (low/high), presentation format (numeric/numeric and qualitative/absolute difference and qualitative/full) and no‐information and qualitative only (online questionnaire)	2531 (18 to 40 y, n = 617; 41 to 52 y, n = 649; 53 to 64 y, n = 643; 65 + y, n = 622)			15
Sutherland et al 1991^78^	Test‐retest study, factorial design. Between groups, two factors: mode of delivery (paper/online), interviewer (1/2) (paper and online questionnaire)	100 (n = 35 per group; interviewer 1, computer administration mean age 54.0 y, SD = 13.3; interviewer 1, paper administration mean age 52.3 y, SD = 13.4; interviewer 2, computer administration mean age 58.7 y, SD = 14.4; interviewer 2, paper administration mean age 57.3 y, SD = 11.6)	Blood transfusions, hypothetical situation	Out‐patients with an established diagnosis of cancer at the Princess Margaret Hospital Lodge (Ontario, Canada)	8
Tan et al 2005^79^	Cross‐sectional study (paper questionnaire), factorial design. Between groups, one group: presentation format (probability/frequency) (paper questionnaire)	Graduate students, n = 38. Health‐care professionals, n = 47. (Mean age, probability format—33.8 y, frequency format—34.2 y)	Influenza vaccine, hypothetical situation	Graduate students attending a biostatistics lecture given by the first author. Group of health‐care professionals attending a workshop given by second author (Singapore)	12
Taylor et al 2007^80^	Cross‐sectional study (postal questionnaire)	1202 (mean age 52.7 y, SD = 6.4, range 18 to 97 y)	Non‐prescription medications, mixed—presented as hypothetical situation, but 42.3% had previously bought a non‐prescription medication at a convenience store, 91.6% had previously bought a non‐prescription medication at a pharmacy	Random sample of adults (Saskatoon, Saskatchewan, Canada)	8
Thorens et al 2008^81^	Cross‐sectional study (face‐to‐face interview)	100 (age 19 to 39 y, n = 58; age 40 and above, n = 42; range 19 to 65 y)	Psychopharmacotherapy, actual situation	Patients in an inpatient ward of the public psychiatric hospital, with a minimum two day stay and with partial remission of acute behavioural or psychotic symptoms (Geneva, Switzerland)	15
Wallace 1985^82^	Study 1: Prospective cohort study (face‐to‐face interview)	118 (not reported)	Post‐surgical pain, actual situation	Female adults undergoing laparoscopic surgery for sterilization or infertility investigation admitted to a gynaecology ward (not reported)	5
	Study 2: Prospective cohort study (face‐to‐face interview)	63 (not reported)			5
Walmsley et al 1992^83^	Cross‐sectional study (face‐to‐face interview)	101 (over 55 y)	Post‐surgical pain, actual situation	Patients scheduled for elective surgery; cardiac patients were excluded (not reported)	10
Webster et al 2017^84^, ^b^	Cross‐sectional study (online questionnaire)	1003 (median age 41.0 y, inter‐quartile range 22.0)	Unnamed drug, hypothetical situation	Random sample of adults aged between 18 and 65 y (England)	19
Webster et al 2017^85^, ^b^	Cross‐sectional study, factorial design. Between groups, one group: severity of side‐effects (mild/severe). Between groups: presentation format (online questionnaire)				
Whitford and Olver 2012^86^	Prospective cohort study (paper questionnaire)	45 (mean age 55.4 y, SD = 13.7, range 22 to 79 y)	Chemotherapy, actual situation	Chemotherapy‐naïve patients in a medical oncology department who had a cancer diagnosis and a prognosis of more than six months (Royal Adelaide Hospital Cancer Center, Australia)	
Woloshin et al 1994^87^	Cross‐sectional study, factorial design. Within‐group, one factor: severity of side‐effect (minor/major) (paper questionnaire)	307 (mean age 36.2 y)	Medication for hypertension, vaccination, surgery, antibiotic, hypothetical situation	Patients seen in a university‐based family practice who were aged 18 y or over, or parents of patients aged younger than 18 y who were not presenting for general physical examination (not reported)	13
Woloshin and Schwartz 2011^88^	Parallel‐group randomized trial, five groups: natural frequency, variable frequency, percentage, percentage plus natural frequency, percentage plus variable frequency (online questionnaire)	2944 (mean age 47 y, range 18 to 93 y)	Drugs for heartburn and cholesterol, hypothetical situations	Adult volunteers randomly selected from a professional survey firm's research panel of about 30,000 households (USA)	
Zachariae et al 2007^89^	Prospective cohort study (paper questionnaire diary)	125 (range 18 to 70 y)	Chemotherapy, actual situation	Women receiving standard adjuvant chemotherapy after surgery for breast cancer aged between 18 and 70 y who could read and write Danish (Aarhus University Hospital, Denmark)	8

^a^ These results are from the same group of participants.

^b^ These results are from the same group of participants.

Most studies investigated hypothetical scenarios in which participants imagined they needed a specified medical intervention and made judgments about the possibility of side‐effects based on information given to them (n = 41). Twenty‐five studies investigated real situations, in which participants were going to receive the medical intervention. Six investigated hypothetical situations, but a proportion of participants were taking or about to start taking that medication.

We identified four basic methods in the literature to measure side‐effect expectations (see Supporting Information S2): likelihood of side‐effects using a Likert‐type scale (n = 26); probability of side‐effects as a percentage (n = 9); frequency of side‐effects as a number (eg out of 100 taking the medication, n = 6); or visual analogue scale (n = 4). The remainder of studies used a combination of these methods.

Forty‐six studies were cross‐sectional, with 36 using a factorial design. Sixteen studies used prospective cohort designs; nine were randomized controlled trials.

### 3.2 Risk of bias

**FIGURE 2:**
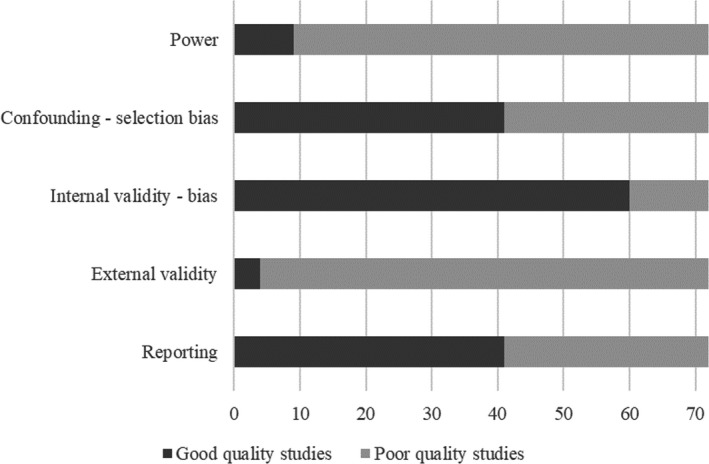
Figure showing number of poor and good quality studies for each aspect of risk of bias

Scores for journal articles ranged between two and 19 out of 19 (see Table 1). Two conference abstracts had artificially low scores (two and four^52^, ^75^). Most studies (n = 34) were poor quality; 32 were moderate quality; and six were good quality. Studies scored particularly poorly for external validity, with only four studies being good quality,^71^, ^74^, ^84^, ^85^ and power, with nine studies being adequately powered^57^, ^60^, ^66^, ^74^, ^77^, ^80^, ^84^, ^85^ (see Figure 2). Thirty‐one studies scored poorly for reporting,^27^, ^29^, ^30^, ^32^, ^37^, ^38^, ^40^, ^41^, ^42^, ^47^, ^50^, ^52^, ^53^, ^54^, ^55^, ^56^, ^62^, ^63^, ^64^, ^65^, ^69^, ^74^, ^75^, ^78^, ^80^, ^82^, ^83^, ^89^ and 31 scored poorly for confounding (selection bias).^28^, ^29^, ^33^, ^34^, ^36^, ^40^, ^41^, ^42^, ^43^, ^44^, ^46^, ^47^, ^52^, ^53^, ^54^, ^57^, ^58^, ^62^, ^63^, ^64^, ^65^, ^70^, ^73^, ^74^, ^75^, ^78^, ^82^, ^89^ Twelve studies scored poorly for internal validity (bias).^42^, ^52^, ^53^, ^56^, ^62^, ^74^, ^75^, ^78^, ^80^, ^82^

### 3.3 Predictors of side‐effect expectation

Results from adjusted and unadjusted analyses are reported together in the text. Where studies reported both adjusted and unadjusted analyses, only adjusted results are reported narratively. Only results from good and moderate quality studies are reported narratively; poor quality studies are reported in summary tables. We evaluated strength of evidence on a case‐by‐case basis to take into account study design. Where study design was the same, we used the following quantifications for the strength of evidence. ‘Good evidence’ was used when 80% or more of the studies investigating a factor found an association. ‘Some evidence’ was used when 60% to 80% of studies investigating the factor found an association. Where all studies found an association, but only few studies investigated an association, we also used the term ‘some evidence.’ ‘No evidence’ was used when less than 60% of studies found an association to account for the effect of publication bias.

#### 3.3.1 Personal characteristics

There was no evidence that gender was associated with side‐effect expectations (see Table 2). Of seven studies, two found an association between female gender and greater side‐effect expectations.^38^, ^51^ One study found an association for two of five outcomes,^84^, ^85^ whereas another found an association between female gender and increased estimates of the probability of side‐effects, but not increased likelihood of side‐effects.^35^ Three studies found no evidence for an association.^71^, ^72^, ^87^

**TABLE 2:** Summary of citations investigating the association between personal characteristics and increased side‐effect expectations; different studies are separated by semi‐colons

	Unadjusted			Adjusted		
	Association	Mixed association	No association	Association	Mixed association	No association
Female gender	(51)	(35)	(28, 29) study 2; (30) study 1; (30) study 2; (30) study 3; (72) study 2; (87)	(38)	(84, 85)	(71)
Male gender		(71)				
Older age	(30) study 1; (30) study 2; (51);(63);(71);		(28, 29) study 2; (30) study 3; (29, 32) study 1; (35);(72) study 2; (87)	(51);(52)	(71)	(38);(84, 85)
Younger age	(50)	(72) study 1			(73)	
Higher level of education	(51)		(28, 29) study 2; (35);(71);(72) study 2; (63)		(73)	(38);(49);(71)
Lower level of education		(72) study 1; (87)			(84, 85)	
White ethnicity (compared to non‐white)			(73);(81);(87);	(38)		
Ethnic minority (compared to white)					(84, 85)	
Born overseas			(71)			(71)
Employed (compared to not employed)						(84, 85)
Occupation (health care–related compared to not‐health care–related)		(87)				
Student or patient (compared to medical doctor)	(34) study 1; (34) study 2	(69)				
Poorer numeracy	(77) benefit design study; (77) risk design study	(44)		(77) benefit design study; (77) risk design study;(40)^a^		
Subjective numeracy						(40)
Menopausal status			(48) study 1; (48) study 2			

^a^ Direction of association not reported.

There was no evidence for an effect of age on side‐effect expectations, with studies reporting mixed findings. Of nine studies, one found an association between older age and increased side‐effect expectations^51^; another found an association between older age and expectations of serious, but not mild side‐effects.^71^ One study found mixed evidence for an association between younger age and side‐effect expectations for nausea, but not vomiting,^72^ while another found an association between younger age and expectations for pain, but not fatigue.^73^ Five studies found no evidence for an association between age and side‐effect expectations.^35^, ^38^, ^72^, ^84^, ^85^, ^87^

There was no evidence for the effect of education on side‐effect expectations, with studies reporting mixed findings. Of nine studies, one found an association between higher education and increased side‐effect expectations,^51^ while another found an association between higher education and increased expectations about the likelihood of fatigue, but not pain.^73^ Three studies found mixed evidence for an association between lower education and increased side‐effect expectations, with one study finding an association for one of five outcomes^84^, ^85^; another finding an association with expected nausea, but not vomiting^72^; and the last finding an association with minor, but not major, complications.^87^ Four studies found no evidence for an association.^35^, ^38^, ^71^, ^72^

There was no evidence for the effect of ethnicity on side‐effect expectations, with studies reporting mixed findings. One study found evidence that people of white ethnicity gave increased estimates of the likelihood of side‐effects compared to non‐white ethnicities.^38^ Conversely, one study found evidence that ethnic minorities gave increased estimates of the likelihood of side‐effects compared to white ethnicities for four of five outcomes.^84^, ^85^ Three studies found no evidence for an association between ethnicity and side‐effect expectations.^73^, ^81^, ^87^ One Australian study found no association with being born overseas and side‐effect expectations.^71^

Studies investigating associations between side‐effect expectations and employment and job role were heterogenous, providing no evidence for an association. One study found that students estimated that a higher percentage of people would experience side‐effects from over‐the‐counter medications than doctors.^34^ Another study found mixed evidence that patients estimated a higher frequency of side‐effects than doctors.^69^ One study found that people who considered their job to be health care–related estimated a higher frequency of side‐effects than those who did not.^87^ Another study found no association between side‐effect expectations and employment.^84^, ^85^

Both studies investigating the association between poorer numeracy and increased side‐effect expectations found an association,^77^ with a third study finding mixed evidence for an association between poorer numeracy and increased probability of certain side‐effects.^44^ One study also found mixed evidence for an association between poorer health literacy and increased side‐effect expectations.^84^, ^85^

#### 3.3.2 Clinical characteristics

##### Side‐effect characteristics

There was some evidence that side‐effects perceived as being less severe were expected to occur more often (see Table 3). Of five studies, three found an association^34^, ^43^, ^87^; two studies found no evidence for an association.^28^, ^29^, ^84^, ^85^ All studies used experimental designs.

**TABLE 3:** Summary of citations investigating the association between clinical characteristics and increased side‐effect expectations; different studies are separated by semi‐colons

	Unadjusted			Adjusted		
	Association	Mixed association	No association	Association	Mixed association	No association
Decreased side‐effect severity (eg milder compared to more severe)	(34) study 1; (34) study 2; (43);(63);(87)		(28, 29) study 1; (29, 32) study 2; (42)			(40);(84, 85)
Increased side‐effect severity (eg more severe compared to milder)		(84, 85)				
Increased likelihood or frequency of side‐effects	(2730) study 1; (29, 32) study 2; (42);(43);(77) risk design study;(78)		(50);(72) study 1; (72) study 2	(30) study 1; (40);(77) risk design study		(38)
Previous experience of symptoms from intervention	(54, 55);(64);(71)	(87)	(80)	(64)	(71);(83)	
No previous experience of intervention			(48) study 1; (48) study 2		(73)	
History of illness			(48) study 1; (48) study 2			
Family history of illness						(73)
Lower effectiveness of medication	(49)	(77) benefit design study; (77) risk design study	(37)	(49)	(77) benefit design study; (77) risk design study	(38)
Existing physical symptoms	(48) study 2	(64)	(47);(48); study 1; (50);(89)	(51);(52)	(64);(73)	(49);(83)
Increased severity of existing physical symptom	(51)					(49)
History of nausea and vomiting in past experiences (eg pregnancy, motion sickness, anxiety).		(64)	(50)			(64)
Lower pre‐treatment quality of life/ worse general well‐being/ health status	(49);(51)			(39)	(84, 85)	(38)
Increased disease severity	(30) study 1		(37)	(30) study 1		

There was no evidence that increased objective likelihood or frequency of side‐effects was associated with increased side‐effect expectations. Of five studies, two experimental studies found an association with increased perceived likelihood of side‐effects.^43^, ^77^ Three studies (one experimental, two longitudinal) found no evidence for an association.^38^, ^72^

##### Previous experience with illness or treatment

There was no evidence that previous experience of a treatment or illness was associated with increased side‐effect expectations. One cross‐sectional study found that previous experience of surgery for breast cancer was associated with decreased expectations for post‐surgical fatigue, but found no evidence for an association with post‐surgical pain.^73^ Two experimental studies found no evidence that previous experience of endocrine treatment, or history of illness, were associated with increased side‐effect expectations.^48^

There was some evidence that previous experience of symptoms from a medical intervention was associated with increased side‐effect expectations. Of three studies, one longitudinal study found an association between having previously experienced side‐effects from the treatment and increased side‐effect expectations.^64^ Two studies (one cross‐sectional, one experimental) found an association with previous side‐effects for mild, but not severe, side‐effect expectations.^71^, ^87^ Another cross‐sectional study found no evidence that knowing more side‐effects from endocrine therapy (free recall, before being given study treatment information) was associated with increased side‐effect expectations.^49^

##### Intervention characteristics

There was some evidence that decreased medication effectiveness (perceived and stated) was associated with increased side‐effect expectations. Of four studies, one cross‐sectional study found an association between decreased medication effectiveness (perceived) and increased side‐effect expectations,^49^ two experimental studies found mixed evidence (stated effectiveness),^77^ and one experimental study found no evidence for an association (perceived effectiveness).^38^ Another experimental study found an association between including extra information about the effectiveness of the treatment and decreased side‐effect expectations.^76^

##### Current symptoms and quality of life

There was some evidence that current experience of symptoms was associated with increased side‐effect expectations. Of six studies, two (one experimental, one longitudinal) found an association between existing physical symptoms and increased side‐effect expectations.^48^, ^51^ One longitudinal study found evidence for an association at one of four timepoints investigated,^64^ while a cross‐sectional study found that pre‐surgical fatigue was associated with increased expectations of post‐surgical fatigue; there were no associations with pre‐surgical pain.^73^ Two studies (one experimental and one cross‐sectional) found no evidence for an association.^48^, ^49^ Two studies investigated the severity of existing symptoms with relation to side‐effect expectations, with one longitudinal study finding an association between increasing severity of existing symptoms and increased side‐effect expectations^51^ and one cross‐sectional study finding no evidence for an association.^49^

There was some evidence that lower pre‐treatment quality of life was associated with increased side‐effect expectations, with two cross‐sectional studies finding an association.^39^, ^49^ Another longitudinal study found an association between worse general well‐being and increased side‐effect expectations.^51^ An experimental study found evidence for an association between chronic illness and increased side‐effect expectations for two of five outcomes.^84^, ^85^ One experimental study found no association between health status and side‐effect expectations.^38^

#### 3.3.3 Psychological traits and state

##### Anxiety and other traits

There was some evidence that heightened health anxiety was associated with increased side‐effect expectations (see Table 4), with one experimental study finding an association.^84^, ^85^ Another longitudinal study found evidence that an anxious preoccupation cancer coping style was associated with increased likelihood and severity of expectations for multiple side‐effects.^86^ There was no evidence that higher trait and state anxiety were associated with increased side‐effect expectations. Two studies (one experimental and one cross‐sectional) found an association between increased trait anxiety and side‐effect expectations,^48^, ^73^ while two studies (one experimental, one longitudinal) found no evidence for an association.^48^, ^64^ One longitudinal study found no evidence for an association between state anxiety and side‐effect expectations.^86^

**TABLE 4:** Summary of citations investigating the association between psychological traits and state and increased side‐effect expectations; different studies are separated by semi‐colons

	Unadjusted			Adjusted		
	Association	Mixed association	No association	Association	Mixed association	No association
Higher health anxiety	(30) study 1; (30) study 2; (50)		(28, 29) study 2; (30) study 3	(84, 85)		
Higher trait anxiety	(48) study 1	(64);(89)	(47);(48) study 2; (54, 55)	(73)		(64)
Higher state anxiety	(82) study 1		(54, 55);(86)			
Higher depression/ combined anxiety and depression score	(49)		(47)			(49)
Optimism						(73);(84, 85)
Negative beliefs about medicines—overuse	(49)			(49)	(84, 85)	
Negative beliefs about medicines—harm			(49)		(84, 85)	
More concerns about treatment than beliefs about its necessity	(49)		(48) study 1; (48) study 2			(49)
Negative beliefs about illness or pain		(49)			(83)	(49)
Increased fear of intervention/distress before intervention	(82)	(65)		(73)		
Increased somatosensory amplification	(89)		(49)			
Increased decisional conflicts about treatment	(48) study 1		(48) study 2			
Style of dealing with medical information		(48) study 2	(48) study 1			

There was no evidence that other psychological traits were associated with side‐effect expectations. One cross‐sectional study found no evidence for an association between combined depression and anxiety score and side‐effect expectations.^49^ Another longitudinal study found no evidence for an association between emotional distress and side‐effect expectations.^64^ Two studies (one experimental and one cross‐sectional) investigated the association between optimism and side‐effect expectations, neither finding evidence for an association.^73^, ^84^, ^85^

There was some evidence that pre‐intervention distress was associated with side‐effect expectations. One cross‐sectional study found that pre‐surgical distress and fear were associated with increased expectations of side‐effects from surgery.^73^ Decisional conflicts about treatment were associated with increased likelihood and severity of side‐effect expectations in one longitudinal study (64 study 1), but not another (64 study 2).

##### Beliefs about medicines

Few studies investigated the association between beliefs about medications and side‐effect expectations, with mixed results. There was some evidence that negative beliefs about the overuse of medications were associated with increased side‐effect expectations, with one cross‐sectional study finding an association^49^ and one experimental study finding an association for four of five outcomes.^84^, ^85^ There was no evidence for an association between negative beliefs about harm that medications could cause and side‐effect expectations, with an experimental study finding an association for four of five outcomes ^84^, ^85^; another cross‐sectional study found no evidence for an association.^49^ There was no evidence that more concerns about medications compared to beliefs about their necessity were associated with side‐effect expectations, with three studies (two experimental and one cross‐sectional) finding no evidence for an association.^48^, ^49^

There was some evidence that increased perceived sensitivity to medicines was associated with increased side‐effect expectations, with one experimental study finding an association.^84^, ^85^ Using a monitoring coping style to deal with illness was associated with increased likelihood and severity of side‐effects in one longitudinal study (64 study 2), but not another (64 study 1). There was no evidence for an association between side‐effect expectations and somatosensory amplification (cross‐sectional),^49^ or social desirability (longitudinal).^64^

#### 3.3.4 Presentation format

##### Verbal and numerical presentation

There was good evidence that describing the incidence of side‐effects verbally, using words such as ‘often’ or ‘rarely’, was associated with greater side‐effect expectations than when describing incidence numerically, using percentages or natural frequencies (see Table 5). Of eight studies, five found an association.^28^, ^29^, ^35^, ^38^, ^57^, ^77^ Two studies found mixed evidence for an association;^45^, ^77^ one study found no evidence for an association.^26^ Two studies found that using only verbal descriptors led to greater expectations of likelihood and severity of side‐effects than using combined verbal and numerical descriptors.^77^ Two studies investigated the use of combined numerical and verbal information, compared to just numerical information. One study found mixed evidence for an association between combined numerical and verbal information and increased side‐effect expectations,^60^ while the other found no evidence for an association.^66^ Another study found that the order of the verbal descriptors of incidence (eg presenting side‐effects which ‘often’ or ‘rarely’ occurred first) did not affect side‐effect expectations.^28^, ^29^ All studies used experimental designs.

**TABLE 5:** Summary of citations investigating the association between presentation format of information and increased side‐effect expectations; different studies are separated by semi‐colons

	Unadjusted			Adjusted		
	Association	Mixed association	No association	Association	Mixed association	No association
Verbal probability statement (eg ‘often’), compared to numerical probability statement (percentage/natural frequency/both)	(28, 29) study 1; (29, 32) study 1; (29, 32) study 2; (35);(56);(57);(58) study 2; (77) risk design study	(58) study 1; (46); (77) benefit design study	(26)	(35);(38); (77) risk design study	(45); (77) benefit design study	
Verbal probability statement (compared to combined numerical and verbal information)	(77) risk design study; (77) benefit design study			(77) risk design study; (77) benefit design study		
Combined numerical and verbal information (compared to just numerical information)		(60)	(66)			
Frequency format (compared to percentage and combined frequency and percentage)	(88)	(59)				
Probability (percentage) format (compared to frequency)	(79)					
Response format (percentage or natural frequency)			(28, 29) study 1			(38)
Only verbal descriptor (eg ‘more’ compared to no information)	(77) benefit design study; (77) risk design study			(77) benefit design study; (77) risk design study		
Personalized information (compared to non‐personalized)			(43)	(48) study 1		(48) study 2
Non‐personalized information (compared to personalized)	(31) study 1; (31) study 2					
Negatively framed information		(67, 68)		(48) study 1		(48) study 2
Verbal qualifier		(41)	(60)			
Narrative summary of information about drugs (compared to facts about drugs	(74) symptom drug box; (74) prevention drug box					
Additional information about medical intervention/receiving supporting therapy			(82) study 2; (75)			

There was no firm evidence for the type of numerical predictor most associated with increased side‐effect expectations. One study found evidence that incidences presented as natural frequencies (eg ‘affects 1 in 50 people’) led to greater estimates of the likelihood of side‐effects than percentages and combined natural frequencies and percentages;^88^ another study found very little (one of seven outcomes) evidence for this association.^59^ One study found that there was a wider spread in the verbal labels assigned by participants to incidences described as percentages than natural frequencies.^79^ One study found that estimated percentages of incidence of side‐effects were greater when communicated as an increase in the number needed to harm (eg ‘for every 500 women…one additional woman will have’) and relative risk (eg ‘the risk…is doubled’) than when communicated as an increase in absolute risk (eg ‘the risk…is 0.02% higher’) in situations with no information about the baseline rate of people affected by that side‐effect.^36^ Two studies found no evidence that the response format (percentage or natural frequency) for estimates of side‐effect expectations affected outcomes.^28^, ^29^, ^38^ All studies used experimental designs.

##### Framing information

There was no evidence that personalizing information (eg ‘you should take two tablets’ compared to ‘two tablets should be taken’) was associated with side‐effect expectations, with studies reporting mixed findings. Of five studies, two found that non‐personalized information was associated with increased expectations of the likelihood of side‐effects.^31^ One study found that personalized information was associated with increased estimates of likelihood and severity of side‐effects.^48^ ^study 1^ Two studies found no evidence for an association between personalized information and side‐effect expectations.^48^ ^study 2,^ ^43^ All studies used experimental designs.

There was some evidence that negatively framed information was associated with increased side‐effect expectations. Of three studies, one experimental study found an association,^48^ ^study 1^ while another longitudinal study found evidence for an association for three out of four outcomes;^67^, ^68^ one experimental study found no evidence for an association.^48^ ^study 2^

Four studies investigated the effect of individual statements on side‐effect expectations. Participants in one study gave higher estimates for the incidence of side‐effects when the baseline rate of side‐effects was not communicated (compared to communicated).^36^ Two studies found that using a verbal descriptor (‘more people had bone loss’) increased side‐effect expectations compared to giving no information about medication effectiveness or side‐effect incidence.^77^ One study found no evidence that using a verbal qualifier (eg ‘will affect’ compared to ‘may affect’) was associated with side‐effect expectations.^60^ All studies used experimental designs.

#### 3.3.5 Information sources

There was no evidence that the number of sources used to gain information about a medical intervention was associated with increased side‐effect expectations, with studies reporting mixed results. One poor quality cross‐sectional study found an association;^52^ as this was a conference abstract, the quality rating score was artificially low. This study also found that using the internet, the National Cancer Institute and American Cancer Society as sources of information about cancer were associated with increased side‐effect expectations, whereas consulting newspapers and primary care physicians were associated with decreased side‐effect expectations.^52^ Another cross‐sectional study found no evidence for an association between number of sources used to gain information about the intervention and side‐effect expectations.^49^ How often participants read patient information leaflets when taking a new medication was also not associated with side‐effect expectations (experimental study).^84^, ^85^ One longitudinal study found that using more media sources to gain information about an illness and its treatment was associated with stating that treatment side‐effects were more likely.^51^

## 4 DISCUSSION

Fear of side‐effects is one of the most commonly cited reasons for not adhering to medical interventions.^9^ Side‐effect expectations have also been associated with decreased intention to adhere to medications.^48^ Side‐effects from medical interventions may not be directly attributable to the treatment itself, but may instead arise through a psychological phenomenon known as the nocebo effect, whereby expectation that an intervention will cause side‐effects is self‐fulfilling.^6^, ^7^, ^8^, ^9^, ^90^ Identifying psychosocial factors associated with side‐effect expectations enables these factors to be targeted by future interventions. Personal and clinical characteristics associated with side‐effect expectations can help identify populations which may be particularly vulnerable to inaccurate side‐effect expectations. This is the first systematic review to synthesize evidence investigating factors affecting side‐effect expectations. Our review identified five broad categories of factors that have been investigated with relation to side‐effect expectations from medical interventions: personal characteristics; clinical characteristics; psychological traits and state; presentation format of information; and information sources used to gain information about the illness and medical intervention.

Clinical characteristics of the medical intervention seem to play a role in influencing side‐effect expectations. There was no evidence that previous experience of a medical intervention was, in itself, associated with increased side‐effect expectations. However, there was some evidence that increased side‐effect expectations were associated with previous experience of side‐effects, in particular mild side‐effects. This corresponds with experimental evidence suggesting that learning about side‐effects can increase expectations and nocebo responding.^5^, ^91^ While more rigorous research is needed to quantify the effect of learning in clinical populations, practitioners should take particular care with patients who have previously experienced side‐effects from treatment. There was some evidence that current experience of symptoms was associated with increased side‐effect expectation, indicating that people may misattribute symptoms to a medical intervention; a key component of the nocebo response.^5^ Results also indicated that factors contributing to overall negative beliefs about the medical intervention, such as being less effective, were associated with increased side‐effect expectations. This is in line with the ‘halo effect’, where attitudes towards dimensions which are perceived as being logically related influence ratings of other dimensions.^92^

Interestingly, only a minority of studies investigating the objective frequency of side‐effects (eg comparing ‘uncommon’ to ‘common’; or ‘1 in 100’ to ‘1 in 10’) found that side‐effect expectations increased in line with objective descriptors. This may be due to strong preconceptions about medication side‐effects which were not influenced by study information, or, where information was presented numerically, because people did not understand the information presented to them due to poor numeracy.^93^ Decreased numeracy is often associated with having less accurate perceptions about the risk of medical interventions^94^ and being more easily influenced by the way numerical information is framed.^95^, ^96^ While only investigated by few studies in this review, poorer numeracy and health literacy were associated with increased side‐effect expectations.

Changing the phrasing of current patient information leaflets may be one of the cheapest ways to alter side‐effect expectations.^97^ Consistent with other research, we found that side‐effect expectations were higher when incidence was described verbally rather than numerically.^98^ However, there was no clear evidence for the type of numerical descriptor (eg percentage or natural frequency) which generated the lowest side‐effect expectations.^99^ Studies investigating the accuracy of side‐effect expectations arising from information presented in different formats have found that using simple infographics, such as pictographs, increases accuracy of estimates of incidence of side‐effects.^100^, ^101^ Pictographs are also perceived as being more trustworthy and helpful than information presented in tables and text.^100^ In addition to presenting information numerically, improving the readability of patient information leaflets, by making the font larger, using simple language and including more pictures,^102^, ^103^ might also increase accuracy of understanding of information about medical interventions.

Very little research has investigated the role of sources of information on side‐effect expectations, with mixed findings. While other research has focused on the negative role of the media on side‐effect reporting,^104^, ^105^ one study included in the review found that consulting newspapers as a source of information was associated with decreased side‐effect expectations.^52^ Research has indicated that side‐effect expectations mediate the association between increased suggestion of side‐effects from different sources, and later perception of side‐effects.^9^ It is therefore important to quantify the role of suggestions from different sources such as online searches, social media, news and the influence of friends, family and health‐care practitioners across different treatments for different illnesses.

There was very little evidence for the influence of psychological traits or state on side‐effect expectations. Increased health anxiety was associated with increased side‐effect expectations, although only one study investigating this factor was good quality. In line with a systematic review finding weak evidence for an association between state and trait anxiety and the nocebo effect, this review found no evidence for an association with increased side‐effect expectations.^5^ Believing that medicines are overused and that you were more sensitive to medicines, were also associated with increased side‐effect expectations; however, few studies investigated these factors. More research is needed to understand how influential wider beliefs about medicines are in the formation of side‐effect expectations.

Evidence from the review indicates that personal characteristics do not systematically influence side‐effect expectations, with studies reporting associations with both increased and decreased side‐effect expectations for multiple factors (eg age, education). Rather than using personal characteristics to target populations for interventions aiming to decrease side‐effect expectations, results of this review suggest that clinical characteristics may be more useful. In particular, clinicians should take care with patients with lower pre‐treatment quality of life and well‐being, those who are currently experiencing symptoms, and those who have previously experienced side‐effects from the treatment.

The aim of this study was to describe the state of the current literature on factors affecting side‐effect expectations. We investigated psychological factors, which could be targeted by interventions, and personal and clinical characteristics, to identify populations that could be at risk of inaccurate expectations. There was inconclusive evidence for most factors investigated. This was likely due to the heterogeneity of studies, with lack of replication of factors in different populations, and the poor quality of studies included. While some factors (eg using verbal compared to numerical descriptors of risk) and populations (eg cancer patients) have been well‐investigated, others have been overlooked. Much research has been carried out in hypothetical situations, in populations who are not about to receive the intervention; future research should concentrate on determining side‐effect expectations in populations about to receive a particular medical intervention. Increased diversity in clinical populations would also allow researchers to identify whether a factor was only influential for a certain medical treatment, or whether it was universally important. Research should be theory‐driven; use standardized measures of assessment (of predictors and outcomes); methodologically rigorous experimental designs; and attempt to replicate results of other studies. Given the growing influence of the media and social media, more research investigating their influence on side‐effect expectations is also needed.

### 4.1 Limitations of the studies included in the review

Most studies included in the review were poor quality. Studies scored particularly poorly for external validity, with only a small number being appropriately powered. Few studies investigated the same predictors; this was particularly notable for studies investigating presentation format. Outcome measures and statistical tests used were also heterogeneous. Studies investigated hypothetical and actual scenarios, with some studies including both people who were due to receive the intervention and those who were not in the same sample. People who were about to receive a medical intervention may have paid more attention to the information given to them about that intervention, or may have interpreted risks differently given the potential for personal experience.^106^

### 4.2 Limitations of the review

Limitations of the review should also be considered. First, studies investigated many side‐effect expectations for many different medical interventions (eg chemotherapy and pills) and in different populations (eg healthy and unwell). We were unable to investigate whether factors were differentially associated with side‐effect expectations for different medical interventions or populations, meaning that we are unable to draw fine‐grained conclusions about whether factors affecting side‐effect expectations differed by medical intervention or study populations. The ecological validity of results, and ability to extrapolate findings to other populations or medical interventions, should be considered when interpreting findings.

Second, few studies investigated the same factors, leading to a lack of replication across studies. Therefore, our interpretation and conclusions for some predictors are based on limited results and should be taken with caution.

Third, we did not search MeSH terms, meaning that we may have missed some studies which were eligible for inclusion.

Fourth, only 19 studies in the review were identified through our search, with the majority coming from reference and forward citation tracking. This may have impacted the results of, and conclusions drawn from, the review.

Fifth, we are aware that any heuristic used in this review to aggregate data (eg counting the number of studies finding significant and non‐significant associations between predictors and side‐effect expectations) are susceptible to bias. More robust methods of reviewing the evidence, such as meta‐analyses, would be preferred to minimize this bias. However, in this case, studies were too heterogeneous to carry out a meta‐analysis.

## 5 CONCLUSION

Clinical characteristics and presentation format may impact side‐effect expectations; there is less evidence for a role of personal characteristics, psychological traits or states, and information sources. There was some evidence that patients who are currently experiencing symptoms; have lower quality of life; and who have previously experienced mild side‐effects from the medical intervention may have heightened side‐effect expectations. Clinicians should take particular care with these patients. Using verbal descriptors of risk, such as ‘common’ or ‘rare’, was associated with greater side‐effect expectations than numerical descriptors, such as percentages or natural frequencies. There was no evidence that a particular type of numerical descriptor was associated with particularly low side‐effect expectations. Widespread, easily‐implementable interventions, such as changing the phrasing and presentation of patient information leaflets and other official communications about medications to use numerical descriptors of risk may lead to decreases in side‐effect expectations, side‐effect perception from medical interventions, and ultimately increase medication adherence. Better quality research, aiming to investigate factors in more varied clinical populations is needed to shed light on whether factors affecting side‐effect expectations are universal to different medical interventions. Research should also attempt to replicate findings, to ensure they are robust.

## CONFLICT OF INTEREST

The authors have no conflicts of interest relevant to this article to disclose.

## Supporting information

Supplementary Material

Supplementary Material

## Data Availability

Data sharing is not applicable to this article as no new data were created or analysed in this study.
