# Supplementary material for: A systematic review of factors associated with side‐effect expectations from medical interventions
Source: Health Expect. 2020 Apr 13;23(4):731–58. doi: 10.1111/hex.13059 (PMC7495066; doi:10.1111/hex.13059)
Supplement: Supplementary file 1 — Supplementary Material [file HEX-23-731-s001.docx]

# Supplementary materials 1. Full search terms and limitations of the search.

Search terms:

- Ovid: ((symptom* or side effect or adverse effect or adverse event or adverse reaction) adj3 expect*)
- Scopus: ((symptom* or {side effect} or {adverse effect} or {adverse event} or {adverse reaction}) w/3 expect*)
- Web of science: (symptom* near/3 expect*) or (side effect near/3 expect*) or (adverse effect near/3 expect*) or (adverse event near/3 expect*) or (adverse reaction near/3 expect*)

Databases used:

- Embase 1974 to 2019 Week 09
- Ovid MEDLINE(R) and Epub Ahead of Print, In-Process & Other Non-Indexed Citations and Daily 1946 to March 05, 2019
- Global Health 1973 to 2019 Week 08
- PsycARTICLES Full Text
- PsycINFO 1806 to February Week 4 2019
- Scopus (until 6^th^ March 2019)
- Web of science (until 6^th^ March 2019)

Limitations of search

- Terms searched keywords, titles and abstracts
- Medical subject headings (MeSH) terms were not searched
- The search was conducted in English only

Exclusion criteria

- Results not published in English were excluded from the review
- Results were not excluded by publication type; any type of publication was permitted
- Results were not excluded by study population; studies were included if they investigated participants of any age, or health status
