# Supplementary material for: A systematic review of factors associated with side‐effect expectations from medical interventions
Source: Health Expect. 2020 Apr 13;23(4):731–58. doi: 10.1111/hex.13059 (PMC7495066; doi:10.1111/hex.13059)
Supplement: Supplementary file 2 — Supplementary Material [file HEX-23-731-s002.docx]

# **Supplementary materials 2**. Full table of results of predictors of increased side-effect expectation (factors significantly associated reported in bold).

| Reference | Symptoms expected | Measure of symptoms expected | Rate of symptom expectation | Predictors of symptom expectation | |
| --- | --- | --- | --- | --- | --- |
|  |  |  |  | Unadjusted | Adjusted [variables controlled for] |
| Al Juffali et al. 2014^26^ | Likelihood of side-effects (dry eyes or loss of hair) from acne medication | “the overall likelihood of their experiencing the side-effect.” Six-point Likert scale.  Cut-off for determining expectation not reported | Dry eyes: verbal probability statement – 46%, numerical probability statement – 41%. Loss of hair: verbal probability statement – 50%, numerical probability statement – 39%. | Likelihood of side-effects: Dry eyes: presentation format. Loss of hair: presentation format. |  |
| Andrykowski & Gregg 1992^27^ | Likelihood of 16 side-effects often associated with chemotherapy | Side-effect Expectancy Questionnaire (SE-EXPECT). Expectations for subsequently experiencing side-effect. Five-point Likert scale: ‘I am certain I will not have this’ (1), ‘unsure’ (3), ‘I am certain I will have this’ (5) | Not reported | **Higher tendency of regimen to cause vomiting** |  |
| Berry et al. 2002^28^; Berry et al. 2002^29^ | Study 1: Probability and frequency of ‘very common,’ ‘common,’ ‘uncommon,’ ‘rare,’ and ‘very rare’ side-effects | Percentage of population who would experience side-effects, number of individuals (out of 10,000) who would experience side-effects | Mean. Very common – 64.7%, common – 44.0%, uncommon – 16.2%, rare – 7.1%, very rare – 3.4% | Order of presentation of probability terms, side-effect severity (mild, severe), type of response (percentage/number out of 10,000). |  |
|  | Study 2: Likelihood and probability of four severe side-effects, specific side-effects varied by experimental condition (one each for throat and ear infection) | ‘How likely do you think it is that you would experience one or more of the side-effects if you took Epidoxin?’ Six-point Likert scale: ‘not at all likely’ (1) to ‘very likely’ (6)  ‘What do you think is the probability that you will experience one or more of these side-effects if you took Epidoxin? Please state your answer as a percentage.’ | Mean. Likelihood of experiencing side-effects: verbal probability statement – 4.4, numerical probability statement – 2.5. Probability of experiencing side-effects: verbal probability statement – 64.4%, numerical probability statement – 20.0%. | Likelihood of side-effects: **verbal probability statement (compared to numerical)**, gender, age, education, health anxiety.  Probability of experiencing side-effects: **verbal probability statement (compared to numerical)**, gender, age, education, health anxiety. |  |
| Berry et al. 2002^30^ | Study 1: Likelihood of side-effects | ‘How likely do you think it is that you would experience one or more of the side-effects if you took Epidoxin?’ Six-point Likert scale: ‘not at all likely’ (1) to ‘very likely’ (6) | Not reported | Gender, **older age, high health anxiety, increased likelihood of side-effects, severe disease (compared to mild)** | Hierarchical regression.  **Increased likelihood of side-effects, severe disease (compared to mild)** [gender, age, education, health anxiety] |
|  | Study 2: Likelihood of side-effects | ‘How likely do you think it is that you would experience one or more of the side-effects if you took Epidoxin?’ Six-point Likert scale: ‘not at all likely’ (1) to ‘very likely’ (6) | Not reported | Gender, **older age, high health anxiety** |  |
|  | Study 3: Likelihood of side-effects | ‘How likely do you think it is that you would experience one or more of the side-effects if you took Epidoxin?’ Six-point Likert scale: ‘not at all likely’ (1) to ‘very likely’ (6) | Not reported | Gender, older age, health anxiety, **perceived** **level of control over side-effects (no statement, alleviation statement;** prevention statement ratings lower**)** | Hierarchical regression. **Level of control over side-effects (no statement, alleviation statement;** prevention statement ratings lower**)**  Severity of side-effects: **severe side-effect (compared to mild) [**gender, age, education, health anxiety] |
| Berry et al. 2003^31^ | Study 1: Likelihood of experiencing one or more of four severe side-effects | How likely do you think it is that you would experience one or more of the side-effects if you took Epidoxin?’ Six-point Likert scale: ‘not at all likely’ (1) to ‘very likely’ (6) | Mean. Likelihood of experiencing side-effects: personalised version of information – 4.18, non-personalised version of information – 4.71 | **Non-personalised version of information (compared to personalised)** |  |
|  | Study 2: Likelihood of experiencing one or more of four severe side-effects | ‘How likely do you think it is that you would experience one or more of the side-effects if you took Epidoxin?’ Six-point Likert scale: ‘not at all likely’ (1) to ‘very likely’ (6) | Mean. Likelihood of experiencing side-effects: personalised version of information – 3.30, non-personalised version of information – 3.85 | **Non-personalised version of information (compared to personalised)** |  |
| Berry et al. 2003^32^; Berry et al. 2002^29^ | Study 1: Probability of experiencing a side-effect | ‘What do you think is the probability that you will experience one or more of these side-effects if you took Epidoxin? Please state your answer as a percentage (e.g. 50%).’ | Range of probability judgements. Verbal descriptor – 5% to 100%, numerical descriptor – 5% to 60%. | Probability of side-effects: **verbal probability statement (compared to numerical),** age |  |
|  | Study 2: Probability of experiencing a side-effect | ‘What do you think is the probability that you will experience one or more of these side-effects if you took Epidoxin? Please state your answer as a percentage (e.g. 50%).’ | Mean estimates of probability judgments. Verbal descriptors: ‘common’ – 50.5%, ‘rare’ – 21.5%. Numerical descriptors: ‘2%’ – 9.5%, ‘0.02%’ – 6.8% | Probability of side-effects: **verbal probability statement (compared to numerical), common frequency (compared to rare),** side-effect severity |  |
| Berry 2004^33^ | Likelihood of one of two sets of mild side-effects | ‘How likely do you think it is that you would experience one or more of the side-effects if you took Epidoxin?’ Six-point Likert scale: ‘not at all severe’ (1) to ‘very severe’ (6) | Mean. Adult patients: Likelihood of side-effects – 4.11. Child patients: Likelihood of side-effects – 4.47. | Likelihood of side-effects: **child patient (compared to adult),** parent status  Severity of side-effects: **child patient (compared to adult),** parent status |  |
| Berry et al. 2004^34^ | Study 1: Probability of either mild or severe side-effects | ‘Participants were required to indicate, for each side-effect, the percentage of the population they would expect to experience the side-effect if they took the medication’ | Mean estimates of probability judgments. ‘Very common’ – 46.5%, ‘common’ – 25.5%, ‘uncommon’ – 7.2%, ‘rare’ – 2.0%, ‘very rare’ – 0.5% | **Student (compared to medical doctors), mild side-effects (compared to severe)** |  |
|  | Study 2: Probability of either mild or severe side-effects | Doctors: ‘participants were required to indicate, for each side-effect, the percentage of the population they would expect to experience the side-effect if they took the medication’  Students: ‘similar to that used in Berry et al [2002a (64)]’ | Overall mean estimates of probability judgments. ‘high >1%’ – 51.0%, ‘moderate 0.1%-1%’ – 28.8%, ‘low 0.01%-0.1%’ – 14.4%, ‘very low 0.001% to 0.0001%’ – 8.9%, ‘minimal 0.0001%-0.00001%’ – 7.7%, ‘negligible <0.00001%’ – 6.6% | **Student (compared to medical doctors), mild side-effects (compared to severe)** |  |
| Berry et al. 2004^35^ | Likelihood and probability of side-effects (stomach discomfort or pain) | ‘How likely do you think it is that you would experience this side-effect if you took the tablets?’ Six-point Likert scale: ‘not at all likely’ (1) to ‘very likely’ (6)  ‘What do you think is the probability that you will experience this side-effect if you took the tablets? Please state your answer as a percentage (e.g. 0, 50, 100%, etc.)’ | Mean. Likelihood of side-effects: verbal descriptor – 3.97, numerical descriptor – 2.61.  Probability of side-effects: verbal descriptor – 56.61%, numerical descriptor – 19.94% | Likelihood of side-effect: **verbal presentation format (compared to numerical),** gender, education, age.  Probability of side-effects: **verbal presentation format (compared to numerical), female gender,** education, age. | Likelihood of side-effect: **verbal presentation format (compared to numerical)** [gender, education, age]  Probability of side-effects: **verbal presentation format (compared to numerical)** [gender, education, age] |
| Berry et al. 2006^36^ | Probability of thromboembolism | ‘From the information provided, what do you think is the risk of having a thromboembolism for women taking the [second/third] generation pill? (Please state you answer as a percentage, where 0% = no women will have a thromboembolism and 100% = every woman will have a thromboembolism.) | Mean. Second generation pill: without baseline – 11.92%, with baseline – 1.39%. Third generation pill: without baseline – 19.32%, with baseline – 2.17%. | Second generation pill: **baseline not communicated (compared to communicated), risk format (baseline not communicated – relative risk and number needed to harm format compared to absolute risk format,** baseline communicated version**).**  Third generation pill: **baseline not communicated (compared to communicated), risk format (baseline not communicated – relative risk and number needed to harm format compared to absolute risk format,** baseline communicated version**).** |  |
| Bersellini & Berry 2007^37^ | Study 2: Belief that the medication causes side-effects | ‘Belief about side-effects.’ Six-point Likert scale: ‘believe medicine is not associated with any side-effects’ (1) to ‘believe it is definitely associated with one or more side-effects’ (6) | Mean – 3.14 | Disease severity, effectiveness statement |  |
| Blalock et al. 2016^38^ | Likelihood of side-effects | ‘If you had high cholesterol and took this medication, how likely is the medication to cause side-effects?’ Seven-point Likert scale: ‘very unlikely’ (0) to ‘very likely’ (6) | Not reported |  | **Side-effect probability format (non-numeric risk format compared to numeric formats),** low and high numeric side-effects, high and low benefit risk difference, age, **female gender, white (compared to non-white participants),** education, health status.  [age, gender, race, education, health status, current medication use, experience of serious medication side-effects] |
| Colagiuri et al. 2008^39^ | Likelihood of post-treatment nausea | Likelihood of nausea, five-point Likert scale: ‘I am certain I will not have this’ (1) to ‘I am certain I will have this’ (5). Expected severity of nausea, six-point Likert scale: ‘very mild or none at all’ (1), ‘mild’ (2), ‘moderate’ (3), ‘severe’ (4), ‘very severe’ (5), ‘intolerable’ (6). Perceived susceptibility to nausea compared to friends and family: ‘more,’ ‘less,’ ‘the same.’ Rate likelihood of nausea compared to other cancer patients: ‘more,’ ‘less,’ ‘the same.’  Expectancy questions combined to give a single expectancy measure. | Not reported |  | **Lower pre-treatment quality of life** [age, gender, susceptibility to motion sickness, diagnosis] |
| Cox 2019^40^ | Study 2: Frequency and likelihood of side-effects | ‘Please indicate the range of users (out of 1,000) who are likely to experience the side-effect after taking this prescription’ Answer: ‘If 1,000 people took this prescription, I would expect between [-] and [-] of these users to experience the listed side-effect.  ‘If I took this drug, I would be likely to experience side-effects.’ Likert-scale |  |  | ANCOVA. Frequency of side-effects: prescriptive pain medications are dangerous, **prescriptive pain medications are addictive,** frequency of news coverage of prescriptive pain medications**, frequency of speaking with friends/family about prescriptive pain medications, objective numeracy (direction not reported),** subjective numeracy, severity, **verbal quantifier** **indicating more common symptom** [prescriptive pain medications are dangerous, prescriptive pain medications are addictive, frequency of news coverage of prescriptive pain medications, frequency of speaking with friends/family about prescriptive pain medications, objective numeracy, subjective numeracy]  Likelihood of side-effects: **prescriptive pain medications are dangerous, prescriptive pain medications are addictive,** frequency of news coverage of prescriptive pain medications, frequency of speaking with friends/family about prescriptive pain medications**, objective numeracy (direction not reported),** subjective numeracy, severity, **verbal quantifier indicating more common symptom** [prescriptive pain medications are dangerous, prescriptive pain medications are addictive, frequency of news coverage of prescriptive pain medications, frequency of speaking with friends/family about prescriptive pain medications, objective numeracy, subjective numeracy] |
| Davis 2007^41^ | Frequency of side-effects from allergy drug (infection, anxiety, insomnia), cholesterol drug (upper respiratory infection, back pain, diarrhoea), insomnia drug (nausea, stomach pain, dizziness) | Frequency of occurrence (1-100) of side-effects. Created summary measures for most severe side-effect, second most severe side-effect, third most severe side-effect by averaging incidence across the three hypothetical drugs. | Not reported | Three side-effect group. Qualifying language (control, if…may, severity/length, **discontinuation** **(less severe side-effect)**, **if…may and severity/length (less severe side-effect),** **if…may and discontinuation (less severe side-effect), severity/length and discontinuation (less severe side-effect), if…may and severity/length and discontinuation (less severe side-effect)**  Six side-effect group. Qualifying language (control, **if…may** **(less severe side-effect)**, severity/length, **discontinuation** **(less severe side-effect)**, **if…may and severity/length (less severe side-effect),** if…may and discontinuation, **severity/length and discontinuation (less severe side-effect), if…may and severity/length and discontinuation (less severe side-effect)** |  |
| Fischer & Jungermann 1996^42^ | Study 1: Frequency of mild side-effects (dry mouth, lack of appetite) or severe side-effects (impaired vision, impaired hearing) | Frequency of occurrence of side-effects. ‘Which numerical interval do you think matches the word "[rarely]"? Subjects were asked for a numerical interval estimate’ | Mean. Mild side-effects: rarely – 8.8%, occasionally – 9.9%, frequently – 31.1%. Severe side-effects: rarely – 2.3%, occasionally – 6.8%, frequently – 23.8%. | **Base rate (100 out of 1000 compared to 10 out of 1000), frequency (more compared to less frequently),** severity of side-effect |  |
| Franic & Pathak 2000^43^ | Probability of mild side-effects (headaches, dry eyes, weight gain) or severe side-effects (seizures, blood clots, gastric ulcers with bleeding) | ‘Provide a single number between 0% to 100% that best describes your opinion of the chance of (you/a woman) experiencing this side-effect if the patient package insert states that: [headaches] occur [rarely]’ | See tables 7-10 in paper | Phrasing, **higher** **frequency of side-effect, context (direction not reported), mild side-effects (see paper for interaction effects)** |  |
| Gardner et al. 2011^44^ | Likelihood of side-effects (hot flushes, cataracts, deep vein thrombosis, pulmonary embolism). Personal probability of experiencing side-effects, probability of average person having any side-effect | ‘From the information you have just read, how likely is it that you would have a side-effect from tamoxifen?’ Six-point Likert scale: ‘not at all likely’ (1) to ‘very likely’ (6).  ‘What do you think is the chance that [you] will have [hot flushes] from taking tamoxifen?’ Answer as percentage | Mean. Likelihood of side-effects – 3.8. | Whole sample. Likelihood of side-effects: numeracy. Probability of side-effects: Hot flushes: numeracy. Cataracts: **poorer** **numeracy**. Deep vein thrombosis: **poorer** **numeracy**. Pulmonary embolism: **poorer** **numeracy**. Any side-effect: numeracy. Average person having any side-effect: numeracy.  Those who have cancer (n=461). Likelihood of side-effects: numeracy. Probability of side-effects: Hot flushes: **poorer** **numeracy**. Cataracts: **poorer** **numeracy**. Deep vein thrombosis: **poorer** **numeracy**. Pulmonary embolism: **poorer** **numeracy**. Any side-effect: numeracy. Average person having any side-effect: numeracy.  Those who have not had cancer (n=130). Likelihood of side-effects: numeracy. Probability of side-effects: Hot flushes: numeracy. Cataracts: numeracy. Deep vein thrombosis: **poorer** **numeracy**. Pulmonary embolism: **poorer** **numeracy**. Any side-effect: numeracy. Average person having any side-effect: numeracy.  Those who have taken/are taking tamoxifen (n=262). Likelihood of side-effects: numeracy. Probability of side-effects: Hot flushes: **poorer** **numeracy**. Cataracts: **poorer** **numeracy**. Deep vein thrombosis: **poorer** **numeracy**. Pulmonary embolism: **poorer** **numeracy**. Any side-effect: numeracy. Average person having any side-effect: numeracy.  Those who have not taken tamoxifen (n=329). Likelihood of side-effects: numeracy. Probability of side-effects: Hot flushes: numeracy. Cataracts: **poorer** **numeracy**. Deep vein thrombosis: **poorer** **numeracy**. Pulmonary embolism: **poorer** **numeracy**. Any side-effect: numeracy. Average person having any side-effect: numeracy. |  |
| Goetsch et al. 1991^47^ | Likelihood of side-effects (more/less depressed or sad than usual, more/less anxious or nervous than usual, more/less tired than usual, more/less pain related to menstruation; headache, backache, other pain) from oral contraceptives. | ‘Do you expect any of the following to occur in the next month?’ ‘Do you think these expected changed, if any, are related to using oral contraceptives?’ Yes/no  Expectancy = total number of negative side-effects expected due to oral contraceptive use | 65% (n=13) participants with no previous contraceptive use expected at least one negative consequence of oral contraceptives.  31% (n=5) participants with previous contraceptive use expected at least one negative change; only two of these five (13%) attributed these expectations to oral contraceptive use | No previous contraceptive use: Depression, trait anxiety, life changes, physical symptoms. |  |
| Heisig et al. 2015^48^ | Study 1: likelihood and severity of three most common side-effects of endocrine therapy | General Assessment of Side-effects Scale (GASE) | Not reported | Necessity-concern balance, **decisional conflicts,** adherence intention, menopausal status, baseline symptoms, **trait** **anxiety,** informational style (blunting, monitoring) history of tumour, experience with cancer treatment. | ANCOVA: **Negatively framed information,** **personalised information** [menopausal status, baseline symptoms, trait anxiety, monitoring, baseline necessity-concern balance] |
|  | Study 2: likelihood and severity of three most common side-effects of chemotherapy | General Assessment of Side-effects Scale (GASE) | Not reported | Necessity-concern balance, decisional conflicts, **decreased** **adherence intention,** menopausal status, **baseline symptoms,** anxiety, **informational style** (blunting, **monitoring**) history of tumour, experience with cancer treatment. | ANCOVA: Negatively framed information, personalised information [menopausal status, baseline symptoms, trait anxiety, monitoring, baseline necessity-concern balance] |
| Heisig et al. 2016^49^ | Likelihood and severity of any side-effects | ‘Please state to which extent you expect to experience side-effects from your endocrine therapy during the first three months of intake.’ Four-point Likert scale: ‘no complaints expected’ (0), ‘mild side-effects expected’ (1), ‘moderate side-effects expected’ (2), ‘severe complaints expected’ (3) | Mean side-effect expectations [0-3] = 0.96 (SD=0.64) | **Negative necessity-concerns balance*,** intention to take adjuvant endocrine treatment, **negative appraisal of adjuvant endocrine therapy, illness perception (consequences,** timeline, personal control, **decreased treatment efficacy, impact of illness on life, concern,** understanding, **emotional response), beliefs about medication (overuse,** harm, benefit**),** somatosensory amplification, **worse quality of life, increased combined anxiety and depression score, increased number of previously known side-effects, increased number of information sources used to gain treatment information,** knowledge of oestrogen receptor status | Multiple regression analysis: education, intensity of pre-existing symptoms, number of pre-existing symptoms, necessity-concerns balance, **negative appraisal of adjuvant endocrine therapy,** **illness perception** (consequences, **decreased treatment efficacy,** impact of illness on life, concern, emotional response), **beliefs about medication (overuse),** combined depression and anxiety score, number of previously known side-effects, number of information sources used to gain treatment information |
| Hickok et al. 2001^50^ | Likelihood of side-effects (post-treatment nausea, vomiting, nervousness, fatigue, sleep problems) from chemotherapy | ‘Expectations of developing five common adverse effects of chemotherapy.’ Five-point Likert scale: ‘I am certain I will *NOT* have this’ (1) to ‘I am certain I *WILL* have this’ (5). Patients who gave a rating of 4 or 5 were said to expect the symptom | 32% (n=20) expected to experience post-treatment nausea | **Anxiety, younger age,** report of pre-treatment nausea, tendency of regiment to cause vomiting, susceptibility to motion sickness |  |
| Hofman et al. 2004^51^ | Likelihood of side-effects (fatigue, hair loss, memory loss, nausea, depression, sleep problems, pain, difficulty concentrating, hot flashes, weight loss, skin problems, shortness of breath) from chemotherapy | ‘For each side-effect, fill in the ONE number that best indicates your expectations. Answer each question based on what you THINK will happen, not on what you HOPE will happen.’ Five point Likert scale: ‘I am certain I WILL NOT have this’ (1) to ‘I am certain I WILL have this’ (5). | Mean side-effect expectations [1-5]: Fatigue – 3.7, hair loss – 3.2, nausea – 2.9, sleep problems – 2.7, weight loss – 2.5, depression – 2.5, skin problems – 2.4, pain – 2.4, hot flashes – 2.4, difficulty concentrating – 2.2,  shortness of breath – 2.2, memory loss – 2.0. | **Increased severity of that symptom (all symptoms), younger age (<60 years), female gender, higher level of education (college or above), worse general well-being, chemotherapy (vs radiation therapy).** | Stepwise linear regression (number of expected side-effects, p-values not reported): **number of baseline symptoms** (strongest predictor), **patient age** (second strongest predictor), **number of media sources** (third strongest predictor), number of medical sources, number of community sources, gender, functional impairment, health score, education. |
| Hofman et al. 2004^52^ | Expectation of twelve common symptoms (not specified) | ‘Surveyed for symptoms they expected from treatment’ | Not reported |  | Stepwise multiple regression. Number of side-effects expected: **number of side-effects already experiencing, older age, number of information sources, source of information (internet, American Cancer Society, National Cancer Institute, primary care physician‖ – associated with lower side-effect expectancy, newspapers‖ – associated with lower side-effect expectancy).** [not reported] |
| Hubal & Day 2006^53^ | Frequency of side-effects | Frequency of side-effects. Numeric task: ‘how often they thought the side-effects would occur… give a ‘ballpark’ number, their best guess about the approximate number of people out of 100 who would experience the side-effect.’ Answer ‘using a scale from 0 (“none”) to 100 (“all”) in steps of 10, with the addition of 0-1 at the low end and 99-100 at the high end.’  Visual task: same instructions given. Answer by placing each frequency term (on a slip of paper) somewhere along a line on a bulletin board to indicate the percentage of cases it represented. From ‘always’ to ‘never.’ | Mean. Frequency of side-effects: numeric task – 49.1, visual task – 48%. Severity of side-effects: numeric task – 60.8, visual task – 58%. | Frequency of side-effects: **Clustering by frequency term used. Groups identified: always (anchor); causes, most, produces, very; a significant proportion, common, frequent, include, is associated with, likely, manifested, many, more, occurs, usual; can, develop, experience, feel, have, may, might, several, somewhat; less, noted, observed, possible, reported, shown, some; a minority of; a few, infrequent; uncommon, unlikely; rare, very rare; never (anchor)** |  |
| Jacobsen et al. 1993^54^† | Likelihood of side-effects (nausea, vomiting, change in taste or appetite, feeling tired, hair loss, skin itching, pain, weakness, diarrhoea, chills, nervousness) from chemotherapy | Five-point Likert scale: ‘certain I will not have this’ (1) to ‘certain I will have this’ (5) | Not reported | Second infusion: Anticipatory anxiety |  |
| Montgomery et al. 1998^55^† | Likelihood of post-treatment nausea from chemotherapy | Adapted Side-effect Expectancy Questionnaire (16 side-effects). 3 Point Likert scale: ‘expect no nausea’ (1), ‘not sure’ (2), ‘expect nausea’ (3) | Not reported | **Higher percentage of infusions followed by nausea, increased severity of post-treatment nausea over first five infusions,** trait anxiety, state anxiety |  |
| Knapp et al. 2001^56^ | Likelihood and probability of side-effects | ‘Rated (on a Likert scale) the likelihood of having the side-effect’  ‘Estimated the percentage of people who would have the side-effect’ | Not reported | Likelihood of side-effects: **verbal probability statement (compared to numerical)**  Probability of side-effects: **verbal probability statement (compared to numerical)** |  |
| Knapp et al. 2004^57^ | Likelihood and probability side-effect (either constipation or pancreatitis) | ‘From the information you have just read, how likely is it that YOU would experience this side-effect from taking [atorvastatin]?’ Six-point Likert scale: ‘not at all likely’ (1) to ‘very likely’ (6).  ‘What do you think is the probability that YOU will experience this side-effect from taking [atorvastatin]? Please state your answer as a percentage.’ | Mean. Verbal descriptor: likelihood – 3.3, probability – 18.0%. Numerical descriptor: likelihood – 2.4, probability – 2.1%. | Likelihood of side-effects: **verbal probability statement (compared to numerical)**  Probability of side-effects: **verbal probability statement (compared to numerical)** |  |
| Knapp et al. 2009^58^ | Study 1: Likelihood and probability of side-effects (sickness and feeling sick, infections, painful muscles and joints, bad allergic reactions) from chemotherapy | ‘How likely they were to experience a side-effect from the medicine,’ Six-point Likert scale: ‘not at all likely’ (1) to ‘very likely’ (6).  ‘Separate estimates of probability that they would experience [sickness and feeling sick, and painful muscles and joints] and any side-effect of Taxol ®.’ Response as a percentage. | Mean. Percent probability statement. Likert items: likelihood of side-effect – 4.04, severity of side-effect – 3.67. Side-effect estimates: sickness or feeling sick – 56.4%, painful muscles or joints – 26.3%, any side-effect – 66.5%. Frequency probability statement. Likert items: likelihood of side-effect – 4.30, severity of side-effect – 3.94. Side-effect estimates: sickness or feeling sick – 52.2%, painful muscles or joints – 29.6%, any side-effect – 66.0%. Verbal probability statement. Likert items: likelihood of side-effect – 4.59, severity of side-effect – 4.15. Side-effect estimates: sickness or feeling sick – 71.6%, painful muscles or joints – 62.2%, any side-effect – 78.8%. | Likelihood of side-effect: presentation format  Probability of developing side-effect: **presentation format (verbal condition compared to frequency and percentage conditions)** |  |
|  | Study 2: Frequency and probability of side-effects (abdominal pain, abdominal upset (sickness and diarrhoea), feeling dizzy, feeling tired) from ibuprofen | Study 2: ‘Separate estimates of probability that they would experience [abdominal pain, abdominal upset, feeling tired] and any side-effect of ibuprofen.’ Half responded as frequency; half responded as percentage. No main effect of response format or interaction with presentation format, therefore conditions were collapsed. | Mean. Percent probability statement. Likert items: likelihood of side-effect – 2.43. Side-effect estimates: abdominal pain – 23.2%, abdominal upset – 16.6%, feeling tired – 12.9%, any side-effect – 23.0%. Frequency probability statement. Likert items: likelihood of side-effect – 2.44. Side-effect estimates: abdominal pain – 14.8%, abdominal upset – 12.5%, feeling tired – 10.7%, any side-effect – 17.8%. Verbal probability statement. Likert items: likelihood of side-effect – 3.48. Side-effect estimates: abdominal pain – 38.5%, abdominal upset – 34.2%, feeling tired – 17.2%, any side-effect – 39.3%. | Likelihood of side-effect: **presentation format (verbal condition compared to frequency and percentage conditions)**  Probability of developing side-effect: Abdominal pain: **presentation format (verbal condition compared to frequency and percentage conditions).** Abdominal upset: **presentation format (verbal condition compared to frequency and percentage conditions).** Feeling tired: presentation format. Any side-effect: **presentation format (verbal condition compared to frequency and percentage conditions).** |  |
| Knapp et al. 2009^45^ | Likelihood and probability of side-effects (hot flushes, cataracts, deep vein thrombosis, pulmonary embolism) from endocrine therapy | ‘From the information you have just read, how likely is it that you would have a side-effect from tamoxifen?’ Six-point Likert scale: ‘not at all likely’ (1) to ‘very likely’ (6).  ‘What do you think is the chance that you will have [hot flushes] from taking tamoxifen? Please state as a percentage in the box below’ | Likelihood of side-effects: verbal descriptor – 4.3, frequency descriptor – 3.6, combined descriptor – 4.3.  Probability of personal chance of any side-effect: verbal descriptor – 72.2%, frequency descriptor – 52.9%, combined descriptor – 42.9%. |  | Likelihood of side-effect: **presentation format (verbal and combined compared to numerical format)**  Probability of developing side-effect: Hot flushes: **presentation format (verbal compared to combined and numerical)**. Cataracts: **presentation format (verbal compared to combined and numerical)**. Deep vein thrombosis: **presentation format (verbal compared to combined and numerical)**. Pulmonary embolism: presentation format. Personal chance of ANY side-effect: **presentation format (verbal compared to combined and numerical)**. [number of side-effects experienced] |
| Knapp et al. 2010^46^ | Probability of experiencing side-effects (hot flushes, cataracts, deep vein thrombosis, pulmonary embolism, any side-effect) from endocrine therapy | ‘What do you think is the chance that [you] will have [hot flushes] from taking tamoxifen?’ Answer as percentage | Hot flushes –54.2%, cataracts – 18.6%, deep vein thrombosis – 12.1%, pulmonary embolism –8.3%, any side-effect – 54.0%, average person having any side-effect – 47.9% | Hot flushes: **type of numerical descriptor (absolute frequency compared to frequency band),** presentation format. Cataracts: **type of numerical descriptor (absolute frequency compared to frequency band),** presentation format. Deep vein thrombosis: type of numerical descriptor, presentation format. Pulmonary embolism: type of numerical descriptor, **presentation format (numeric compared to numeric and verbal descriptors)**. Any side-effect: **type of numerical descriptor (absolute frequency compared to frequency band),** presentation format. Average person having any side-effect: type of numerical descriptor, presentation format. |  |
| Knapp et al. 2013^59^ | Likelihood and probability of side-effects (hot flushes, cataracts, deep vein thrombosis, pulmonary embolism, any side-effect) from endocrine therapy | ‘From the information you have just read, how likely is it that you would have a side-effect from tamoxifen?’ Six-point Likert scale: ‘not at all likely’ (1) to ‘very likely’ (6).  ‘What do you think is the chance that [you] will have [hot flushes] from taking tamoxifen?’ Answer as percentage | Mean. Likelihood of side-effects – 4.2. Median. Probability of side-effects: hot flushes –50.0%, cataracts – 3.0%, deep vein thrombosis – 0.5%, pulmonary embolism – 0.35%, any side-effect – 50.0%, average person having any side-effect – 50.0% | Likelihood of side-effects: presentation format. Probability of side-effects: Hot flushes: presentation format. Cataracts: **presentation format (frequency higher than percentage and combined percentage and frequency)**. Deep vein thrombosis: presentation format. Pulmonary embolism: presentation format. Any side-effect: presentation format. Average person having any side-effect: presentation format. |  |
| Knapp et al. 2016^60^ | Likelihood and probability of side-effects (aching joints and muscles, severe anaemia, serious allergic reaction, itching, dizziness or fits, any side-effect) from chemotherapy | ‘From the information you have just read, how likely is it that you would have a side-effect from Paclitaxel?’ Six-point Likert scale: ‘not at all likely’ (1) to ‘very likely’ (6).  ‘What do you think is the chance that you will have [aching joints and muscles] from taking Paclitaxel?’ Answer as percentage. | Mean. Likelihood of side-effects: verbal and numerical term – 3.8%, numerical term only – 3.4%. Probability of side-effects. Aching joints and muscles: verbal and numerical term – 23.1%, numerical term only – 13.2%. Severe anaemia: verbal and numerical term – 14.7%, numerical term only – 11.2%. Serious allergic reaction: verbal and numerical term – 4.2%, numerical term only – 2.3%. Itching: verbal and numerical term –5.2%, numerical term only – 1.6%. Dizziness: verbal and numerical term – 3.3%, numerical term only – 1.4%. Any side-effect: verbal and numerical term – 31.1%, numerical term only – 18.7%. | All participants. Likelihood of side-effects: **presentation format (verbal and numerical condition compared numerical only),** verbal qualifier. Probability of side-effects: Aching joints and muscles: **presentation format (verbal and numerical condition compared numerical only),** verbal qualifier. Severe anaemia: **presentation format (verbal and numerical condition compared numerical only),** verbal qualifier. Serious allergic reaction: presentation format, verbal qualifier. Itching: **presentation format (verbal and numerical condition compared numerical only),** verbal qualifier. Dizziness or fits: presentation format, verbal qualifier. Any side-effect: **presentation format (verbal and numerical condition compared numerical only),** verbal qualifier.  Participants with cancer. Likelihood of side-effects: presentation format, verbal qualifier. Probability of side-effects: Aching joints and muscles: **presentation format (verbal and numerical condition compared numerical only),** verbal qualifier. Severe anaemia: presentation format, verbal qualifier. Serious allergic reaction: presentation format, verbal qualifier. Itching: **presentation format (verbal and numerical condition compared numerical only),** verbal qualifier. Dizziness or fits: presentation format, verbal qualifier. Any side-effect: **presentation format (verbal and numerical condition compared numerical only),** verbal qualifier. |  |
| Lynch & Berry 2007^61^ | Likelihood of side-effects from over-the-counter medication and prescription medication or herbal remedies. | ‘How likely do you think it is that such medicines would be associated with adverse side-effects?’ Six-point Likert Scale: ‘not at all likely’ (1) to ‘very likely’ (6) | Study 1: Mean. Prescribed – 3.6, over-the-counter – 2.91, herbal – 2.34. | **Medication type: prescription medication more than over-the-counter medications** |  |
| Mapes 1979^62^ | Likelihood of side-effects from beta-blocker, antihistamine chloramphenicol, neomycin sulphate | ‘What does this word rare mean? A series of measures of "rarity" are given below. Please tick the box which the word "rare" conveys to you in this context.’ | Beta blocker: less than 1 per 1000 – 59.4%, between 1 and 10 per 1000 – 31.2%, between 10 and 50 per 1000 – 9.4%, between 50 and 100 per 1000 – 0.0%, greater than 100 per 1000 – 0.0%.  Antihistamine: less than 1 per 1000 – 20.7%, between 1 and 10 per 1000 – 62.1%, between 10 and 50 per 1000 – 17.2%, between 50 and 100 per 1000 – 0.0%, greater than 100 per 1000 – 0.0%.  Chloramphenicol, interpretation of ‘frequent’ side-effects: 10-30% - 9%, 30-50% - 13%, 50-70% - 6%, 70-90% - 1%, <90% - 0%.  Neomycin sulphate, interpretation of ‘frequent’ side-effects: 10-30% - 18%, 30-50% - 6%, 50-70% - 6%, 70-90% - 3%, <90% - 0%. | **Antihistamine compared to beta-blocker.** |  |
| Mazur & Merz 1994^63^ | Probability of rare outcome (death, severe pneumonia) from anaesthesia | ‘Asked to give the numerical estimate that they understood when they heard the probability of that outcome was “rare”.’ | Not reported | Answering “less than” the lower bound of the scale: **scale length (more from long compared to short version).**  “Rare” chance of death: **scale length (direction not reported).**  Probability of outcome: **severity (severe pneumonia compared to death),** education, **older age.** |  |
| Montgomery & Bovbjerg 2003^64^ | Likelihood of post-treatment nausea from chemotherapy | Adapted Side-effect Expectancy Questionnaire (23 side-effects).  Three-point Likert scale: ‘I will have no post-treatment nausea’ (0) to ‘I will have post-treatment nausea’ (2) | Not reported | Demographic and medical variables (not specified)  Infusion 1: concurrent emotional distress, concurrent nausea, lifetime history of nausea and vomiting, **trait anxiety, social desirability**  Infusion 2: infusion 1 expectations, **post-treatment nausea after infusion 1, concurrent emotional distress,** concurrent nausea, **lifetime history of nausea and vomiting,** trait anxiety, social desirability  Infusion 3: infusion 1 expectations, **post-treatment nausea after infusions 1 and 2,** concurrent emotional distress, concurrent nausea, lifetime history of nausea and vomiting, trait anxiety, social desirability  Infusion 4: infusion 1 expectations, **post-treatment nausea after infusions 1-3,** concurrent emotional distress, **concurrent nausea,** lifetime history of nausea and vomiting, trait anxiety, social desirability | Simultaneous regression: Infusion 1: concurrent emotional distress, concurrent nausea, lifetime history of nausea and vomiting, trait anxiety, social desirability  Infusion 2: **post-treatment nausea after infusion 1, infusion 1 expectations,** concurrent emotional distress, concurrent nausea, lifetime history of nausea and vomiting, trait anxiety, social desirability  Infusion 3: **post-treatment nausea after infusions 1 and 2,** infusion 1 expectations, concurrent emotional distress, concurrent nausea, lifetime history of nausea and vomiting, trait anxiety, social desirability  Infusion 4: **post-treatment nausea after infusions 1-3, concurrent nausea,** infusion 1 expectations, concurrent emotional distress, lifetime history of nausea and vomiting, trait anxiety, social desirability |
| Montgomery & Bovbjerg 2004^65^ | Likelihood of pain, nausea, fatigue, discomfort, hearing loss from surgery for breast cancer | Visual analogue scale. ‘After surgery, how much [pain] do you think you will feel? Please put a slash through this line (shown below on the actual forms) to indicate how much [pain] you expect to feel.’ ‘No pain at all’ to ‘as much pain as there could be’ | Mean. Pain – 45.27, nausea – 22.83, fatigue – 57.22, discomfort – 53.71, hearing loss – 29.90 | Pain: surgery type, **pre-surgery distress**. Nausea: surgery type, pre-surgery distress. Fatigue: surgery type, **pre-surgery distress**. Discomfort: surgery type, **pre-surgery distress**. Hearing loss: surgery type. |  |
| Moraes & Dal Pizzol 2018^66^ | Frequency of side-effects (one each of common, uncommon, rare) for gastrointestinal problems | ‘According to the information you read, how many people do you think would have the side-effect [name of the side-effect] from the medicine if 100 people took this medicine? Please provide a number’ | Not reported | Presentation format |  |
| O’Connor et al. 1996^67^; O’Connor et al. 1997^68^ | Probability of side-effects from the influenza vaccine | Probability scales from 0-100% (21 items, both positive and negative outcomes e.g. getting a sore arm and remaining free of a sore arm) | Not reported | Getting systemic side-effects: negatively framed information  Getting local side-effects: **negatively framed information**  Remaining free of systemic side-effects: **positively framed information**  Remaining free of local side-effects: **positively framed information** |  |
| Ohnishi et al. 2002^69^ | Frequency of side-effects from a cold, and anti-cancer drug | ‘Please write in parentheses the number (decimal figure permitted) you think appropriate so that left and right sentences appear equivalent.  It [perhaps] occurs. It occurs in ( ) of 100…’ | See Figure 1 in paper | Certain: **group (patient compared to physician),** medicine. Probably: group, medicine. Perhaps: group, **medicine** **(anti-cancer compared to cold)**. Often: group, **medicine** **(anti-cancer compared to cold)**. Sometimes: group, medicine. Infrequently: **group (patient compared to physician),** medicine. Improbably: **group (patient compared to physician),** medicine. Unlikely: group, medicine. Rarely: group, medicine. Never: **group (patient compared to physician),** medicine. |  |
| Pan et al. 2018^70^ | Severity of side-effects from adjuvant endocrine treatment | General Assessment of Side-effects Scale (GASE) – Expect. Four-point Likert scale: ‘none expected’ (0) to ‘severe’ (3) | Not reported | **More severe side-effects expected at 24 months (compared to baseline).** |  |
| Parrella et al. 2013^71^ | Likelihood of potential adverse reactions from youngest child’s latest vaccination | Mild side-effect. ‘How likely did you think he/she would experience a reaction such as fever, irritability or redness at the injection site?’ Five point Likert scale: ‘very likely’ (1), ‘somewhat likely’ (2), ‘undecided’ (3), ‘not too likely’ (4), ‘not at all likely’ (5)  Severe side-effect. ‘How likely did you think he/she would experience a reaction that would need medical treatment from a hospital or GP?’ Five-point Likert scale: ‘very likely’ (1), ‘somewhat likely’ (2), ‘undecided’ (3), ‘not too likely’ (4), ‘not at all likely’ (5) | Expecting a mild side-effect: very likely – 16.9% (n=75), somewhat likely – 33.6% (n=149), undecided – 1.3% (n=6), not too likely – 33.3% (n=148), not at all likely – 15% (n=66)  Expecting a severe side-effect: very likely – 1.8% (n=8), somewhat likely – 7.2% (n=31), undecided – 0.3% (n=1), not too likely – 39.0% (n=170), not at all likely – 51.7% (n=225) | Experiencing a mild side-effect: **male parent gender,** **older parent age** **(**35-44 years, **45+ years;** reference category 18-34 years**),** born overseas, number of children, level of education, household income, socioeconomic status, **parents’ indication that child had previously experienced an adverse effect from any immunisation**  Experiencing a serious side-effect: parent gender, **older parent age** **(**35-44 years, **45+ years;** reference category 18-34 years**),** born overseas, **number of children** **(2, 3,** 4+; reference category 1**)**, level of education, household income, socioeconomic status, **parents’ indication that child had previously experienced an adverse effect from any immunisation** | Multivariate regression: Experiencing a mild side-effect: parent gender, parent age, born overseas, number of children, level of education**, household income (AUD20,001-40,000,** 40,001-60,000, 60,001-80,000, **80,001-100,000,** >100,000; reference category ≤20,000) socioeconomic status, **parents’ indication that child had previously experienced an adverse effect from any immunisation**  Experiencing a serious side-effect: parent gender, **older parent age** **(35-44 years,** **45+ years;** reference category 18-34 years**),** born overseas, **number of children** **(2, 3,** 4+; reference category 1**)**, level of education, household income, socioeconomic status, parents’ indication that child had previously experienced an adverse effect from any immunisation |
| Roscoe et al. 2000^72^ | Likelihood of nausea and vomiting from chemotherapy | ‘Expectations of developing nausea and vomiting.’ Five-point Likert scale: ‘I am certain I will not have this’ (1) to ‘I am certain I will have this’ (5). Patients who gave a rating of 4 or 5 were said to expect the symptom | Study 1. 31% (n=9) expected to experience treatment-induced nausea. 24% (n=7) expected to experience treatment-induced vomiting. | Nausea: **younger age, fewer years of education,** chemotherapy regimen  Vomiting: age, education, chemotherapy regimen |  |
|  |  |  | Study 2: 32% (n=26) expected to experience treatment-induced nausea. 20% (n=16) expected to experience treatment-induced vomiting. | Nausea: age, gender, education, tendency of regiment to cause vomiting  Vomiting: age, gender, education, chemotherapy regimen |  |
| Schnur et al. 2007^73^ | Likelihood of pain and fatigue from surgery for breast cancer | Visual analogue scale. Pain: ‘After surgery, how much pain do you think you will feel?’ Fatigue ‘After surgery, how fatigued do you expect to be?’ | Not reported | Pain: **younger age,** ethnicity, education, marital status  Fatigue: age, ethnicity, **more years of education,** marital status | Path analysis. Model 1: Pain and fatigue. Pre-surgical mood disturbance, **pre-surgical distress, pre-surgical fatigue, pre-surgical pain, higher trait anxiety**, trait optimism, past breast biopsy, previous same surgery, **family history of cancer, having had the same procedure before (direction not reported)**  Model 2: Pain. **Younger age,** family history of cancer, **higher trait anxiety, pre-surgical distress,** education, pre-surgical pain, pre-surgical fatigue, previous same surgery**.**  Fatigue. Age, family history of cancer, **higher trait anxiety, pre-surgical distress, higher education,** pre-surgical pain, **pre-surgical fatigue, not having had previous same surgery.**  [age, years of education] |
| Schwartz et al. 2009^74^ | Symptom drug box: perceived magnitude of side-effects | Not reported | Not reported | **Control box (compared to drug box)** |  |
|  | Prevention drug box: perceived magnitude of side-effects | Not reported | Not reported | **Control box (compared to drug box)** |  |
| Shedden-Mora et al. 2017^75^ | Severity of side-effects from adjuvant endocrine therapy | General Assessment of Side-effects Scale (GASE) – expect | Not reported | Side-effect prevention training |  |
| Shelke et al. 2008^76^ | Likelihood of nausea from chemotherapy | Five-point Likert scale: ‘I am certain I will not have nausea’ (1) to ‘I am certain I will have nausea’ (5). Patients who gave a rating of 4 or 5 were said to expect the symptom. Measured before and after being given education materials. | Pre-education materials. Expected to have nausea, n=94. Did not expect to have nausea or were unsure, n=226. | **Control group (compared to intervention)** |  |
| Sullivan et al. 2015^77^ | Perceived risk (average of two items assessing likelihood and severity of side-effects) | Likelihood of side-effects. ‘How likely it was that participants would have side-effects on Fentiva.’ Seven-point Likert scale: ‘very unlikely’ (1) to ‘very likely’ (7).  Severity of side-effects. ‘How serious side-effects would be.’ Seven-point Likert scale: ‘not at all serious’ (1) to ‘very serious’ (7) | Benefit design study: Mean. No information – 4.13, qualitative only – 4.67, numeric low benefit – 4.42, numeric high benefit – 3.83, numeric and qualitative low benefit – 4.34, numeric and qualitative high benefit – 3.91, absolute difference and qualitative low benefit – 4.29, absolute difference and qualitative high benefit – 4.00, full low benefit – 4.25, full high benefit – 3.83. | **Low benefit, decreased numeracy, presentation format (qualitative only (compared to no information),** numeric low benefit, **numeric high benefit‡,** numeric and qualitative low benefit, **numeric and qualitative high benefit‡ ,** **absolute difference and qualitative low benefit‡, absolute difference and qualitative high benefit‡, full low benefit‡ , full high benefit‡)** | **Low benefit, decreased numeracy, presentation format (qualitative only (compared to no information),** numeric low benefit, **numeric high benefit‡,** numeric and qualitative low benefit, **numeric and qualitative high benefit‡,** **absolute difference and qualitative low benefit‡, absolute difference and qualitative high benefit‡, full low benefit‡, full high benefit‡)** [age, sex, race, ethnicity, educational level, heartburn medication status] |
|  |  |  | Risk design study: Mean. No information – 3.71, qualitative only – 4.41, numeric low risk – 3.37, numeric high risk – 4.01, numeric and qualitative low risk – 3.61, numeric and qualitative high risk – 3.89, absolute difference and qualitative low risk – 3.55, absolute difference and qualitative high risk – 4.00, full low risk – 3.42, full high risk – 3.96. | **High risk, decreased numeracy, presentation format (qualitative only (compared to no information), numeric low benefit‡, numeric high benefit‡, numeric and qualitative low benefit‡,** **numeric and qualitative high benefit‡,** **absolute difference and qualitative low benefit‡, absolute difference and qualitative high benefit‡, full low benefit‡, full high benefit‡)** | **High risk, decreased numeracy, presentation format (qualitative only (compared to no information), numeric low benefit‡, numeric high benefit‡, numeric and qualitative low benefit‡,** **numeric and qualitative high benefit‡,** **absolute difference and qualitative low benefit‡, absolute difference and qualitative high benefit‡, full low benefit‡, full high benefit‡)** [age, sex, race, ethnicity, educational level, heartburn medication status] |
| Sutherland et al. 1991^78^ | Frequency of ‘rare’ side-effects (viruses in blood stream which subsequently cause the patient to feel ill but do not lead to death, viruses in blood stream which subsequently will cause death) | Choose one of the following possibilities: less than 1 per 1000, between 1 and 10 per 1000, between 10 and 50 per 1000, between 50 and 100 per 1000, greater than 100 per 1000. | Means, interviewer 2, paper version. Always – 97.3%, usually – 83.7%, common – 76.4%, frequently – 67.8%, likely – 70.1%, possible –40.6%, may – 37.7%, less common – 21.1%, occasionally – 26.4%, small chance – 13.6%, unusual – 23.0%, unlikely – 29.4%, very rare – 18.1%, rare – 24.6%, never – 18.8%. | **Probability descriptor,** **interviewer** **(not reported)**, questionnaire format, time. |  |
| Tan et al. 2005^79^ | Likelihood of side-effects from influenza vaccine | ‘In your opinion, which of the following phrases best describes a [5%] frequency of side-effect?’ Answers: ‘very common,’ ‘common,’ ‘occasional,’ ‘uncommon, ‘rare,’ ‘very rare’ | All subjects. Probability format. Very common – 7.1%, common – 21.4%, occasional – 26.2%, uncommon – 28.6%, rare – 16.7%. Frequency format. Very common – 2.3%, common –53.5%, occasional – 30.2%, uncommon – 7.0%, rare – 7.0%. | **Presentation format (wider spread in probability format; in frequency format most people endorsed label ‘uncommon’)** |  |
| Taylor et al. 2007^80^ | Likelihood of side-effects from non-prescription medications | ‘I expect over-the-counter medicines to have very few side-effects.’ Seven point Likert scale: ‘strongly disagree’ (1) to ‘strongly agree’ (7) | Bought from a convenience store, mean 5.0. Bought from a pharmacy, mean 5.1 | **Location of sale (convenience store vs pharmacy),** previously reporting an adverse event from non-prescription medication, previous experience of purchasing non-prescription medications from convenience stores (results not reported) |  |
| Thorens et al. 2008^81^ | Likelihood of side-effects from psychopharmacotherapy | ‘Do you expect side-effects?’ Five-point Likert scale: ‘not at all’ (1), ‘not really’ (2), ‘not sure’ (3), ‘rather yes’ (4), ‘yes, very much’ (5). Responses dichotomised (not at all; not really; not sure / rather yes; yes, very much) | Expecting side-effects: Swiss 66.7% (total n=54), European Union 60.0% (total n=24-25), Others 81% (total n=21) | Ethnicity |  |
| Wallace 1985^82^ | Study 1: Likelihood of post-surgical pain | Accuracy of expectations questionnaire | Not reported | Expectations of pain intensity: **state anxiety measured on morning of surgery, fear measured on call for surgery** |  |
|  | Study 2: Likelihood of post-surgical pain | Accuracy of expectations questionnaire | Not reported | Information about surgery (routine care, routine care plus a minimal information booklet, routine care plus a maximal information booklet) |  |
| Walmsley et al. 1992^83^ | Likelihood of post-surgical pain | ‘Thinking about this operation, show me on this scale how much pain you expect to have.’ Visual analogue scale (10 cm): ‘no pain’ to ‘pain as bad as it could be’ | Not reported |  | Stepwise multiple regression (steps not reported). Pre-surgical pain, attitudes about pain (I can handle most pain, doctors take care of pain well, my pain will be relieved, nurses take care of pain well, if I know what pain to expect I can handle it, **pain is to be expected after surgery even with medicine**), pain history – specific previous surgeries, **pain history – all previous surgeries** [not reported] |
| Webster et al. 2017^84^§ | Likelihood of side-effects from hypothetical drug | How likely participants would be if they were to take the drug. Five-point Likert scale: ‘very unlikely (1) to ‘very likely (5) | Answered very likely/likely: very common side-effects 51.9% (n=521), common side-effects 45.0% (n=451), uncommon side-effects 8.1% (n=81), rare side-effects 5.8% (n=58), very rare side-effects 4.1% (n=41) | Very common side-effects: severity of side-effect  Common side-effects: severity of side-effect  Uncommon side-effects: severity of side-effect  Rare side-effects: **severe side-effect (compared to mild)**  Very rare side-effects: severity of side-effect | Stepwise ordinal regressions: Very common side-effects: **female gender,** age, ethnicity, employment status, education, **household illness (**self, **other;** reference category none**),** severity of side-effect, optimism, **perceived sensitivity to medicines,** beliefs about medicines (harm, overuse), health anxiety, health illiteracy, PIL reading frequency, numerical estimate of incidence of side-effect for descriptor  Common side-effects: **female gender,** age, **ethnic minority,** employment status, education, **household illness (self, other;** reference category none**),** severity of side-effect, optimism, **perceived sensitivity to medicines,** **beliefs about medicines (harm, overuse),** health anxiety**, health illiteracy,** PIL reading frequency, numerical estimate of incidence of side-effect for descriptor  Uncommon side-effects: gender, age, **ethnic minority,** employment status, **lower education, household illness (self, other;** reference category none**),** severity of side-effect, optimism, **perceived sensitivity to medicines,** **beliefs about medicines (harm, overuse),** health anxiety, **health illiteracy,** PIL reading frequency, numerical estimate of incidence of side-effect for descriptor (**underestimate,** overestimate; reference category correct)  Rare side-effects: gender, age, **ethnic minority,** employment status, **lower education,** household illness (self, other; reference category none), severity of side-effect, optimism, **perceived sensitivity to medicines,** **beliefs about medicines (harm, overuse),** health anxiety, **health illiteracy,** PIL reading frequency, numerical estimate of incidence of side-effect for descriptor  Very rare side-effects: gender, age, **ethnic minority,** employment status, **lower education,** household illness (self, other; reference category none), severity of side-effect, optimism, **perceived sensitivity to medicines,** **beliefs about medicines (harm, overuse),** health anxiety, **health illiteracy,** PIL reading frequency, **underestimated** **numerical incidence of side-effect for descriptor**  [gender, age, ethnicity, employment status, education, household illness, side-effect type (mild/severe)] |
| Webster et al. 2017^85^§ | Frequency of mild (headache, nausea) and severe (seizure, difficulty breathing) side-effects from hypothetical drug | ‘Consider a PIL for an unnamed drug that stated, for example, that ‘nausea is common’. Items then asked participants to estimate how many out of 10,000 people who take the drug would develop that side-effect.’ | Mild side-effects. Very common – 84.4% endorsed 1001-10,000 per 10,000 patients, common – 81.2% endorsed 1001-10,000 per 10,000 patients, ‘low risk verbal descriptors’ – 61.8% to 76.7% endorsed 101 to 1000 or 11 to 100 per 10,000. Severe side-effects. ‘High risk verbal descriptors’ – 56.3% to 71.2% endorsed 1001 to 10,000 per 10,000, ‘low risk verbal descriptors’ – 43.4 to 70.6% endorsed 101 to 1000 or 11 to 100 per 10,000. | **Severe side-effects (compared to mild side-effects)** |  |
| Whitford & Olver 2012^86^ | Likelihood and severity of 20 common chemotherapy-related toxicities | ‘Rate the expected severity of each toxicity on linear analogue self-assessment  (LASA) indicators’ from  ‘do not expect the side-effect at all’ (0) to ‘expect the worst possible severity of the side’ (100) | Not reported | State anxiety, **cancer coping style (**fighting spirit, helpless/hopeless, **anxious preoccupation⁋,** fatalistic, avoidance**)** |  |
| Woloshin et al. 1994^87^ | Frequency of side-effects from hypertension medication (fatigue), vaccination (soreness), surgery (nausea), antibiotic (diarrhoea) | ‘Participants estimated how many people of 100 would have each complication or side-effect’ | Hypertension medication: minor complications – 12.3, major complications – 2.8. Vaccination: minor complications – 17.0, major complications – 4.7. Surgery: minor complications – 17.3, major complications – 1.8. Antibiotic reaction: minor complications – 12.5, major complications – 3.5. | **Severity of complications (minor compared to major),** age, gender, ethnicity, **occupation (healthcare related compared to not- only for minor complications), lower education (minor complications,** major complications**), previously had side-effect (minor complications,** major complications**).** |  |
| Woloshin & Schwartz 2011^88^ | Magnitude of side-effects | ‘Overall, how would you describe the side-effects of [PAXCID] for people with bothersome heartburn?’ Five-point Likert scale with additional answer: ‘very large,’ ‘large,’ ‘moderate,’ ‘small,’ ‘very small’ and ‘there are no side-effects’ |  | Heartburn drug. **Presentation format (natural frequency higher than variable frequency, percent, percent plus natural frequency, percent plus variable frequency).**  Cholesterol drug. **Presentation format (natural frequency higher than percent, percent plus natural frequency, percent plus variable frequency,** no difference with variable frequency**)** |  |
| Zachariae et al. 2007^89^ | Likelihood of nausea and vomiting and fatigue | 100mm Visual Analogue Scale | Mean. Women with anticipatory nausea – 40.6, women without anticipatory nausea – 41.7 | Expected nausea: autonomic perception, **somatosensory amplification,** involvement and openness to experience, **trait anxiety,** presence of anticipatory nausea  Expected vomiting: autonomic perception, **somatosensory amplification,** involvement and openness to experience, trait anxiety |  |

* Negative balance indicates more concerns about treatment than beliefs about its necessity

†These results are from the same group of participants

‡ Inverse relationship – lower than qualitative information only

‖ Inverse relationship – associated with lower side-effect expectations

§ These results are from the same group of participants

⁋ See Whitford & Olver 2012 (80), Table 6 for associations with individual symptoms
